# Generative AI in simulation debriefings: an exploratory study using the Team-FIRST framework and qualitative feedback from simulation experts and learners

**DOI:** 10.1186/s41077-026-00407-0

**Published:** 2026-01-29

**Authors:** David W. Tscholl, Max Ebensperger, Arend RahrischRahrisch, Helius Wang, Hubert Heckel, Max Thomasius, Alexander Kaserer, Bastian Grande, Julia C. Seelandt, Michaela Kolbe

**Affiliations:** 1 Institute for Anesthesiology and Perioperative Medicine, University Hospital Zurich, University of Zurich, Zurich, Switzerland; 2 Department of Anesthesiology and Intensive Care, TUM School of Medicine and Health, Technical University of Munich, Munich, Germany; 3 University of Zurich, Zurich, Switzerland; 4 Simulation Center, University Hospital Zurich, Zurich, Switzerland; 5 ETH Zurich, Zurich, Switzerland

**Keywords:** Generative artificial intelligence, Healthcare, Simulation, Debriefing, Large language models, Teamwork, Qualitative thematic analysis, Clinical education technology, Automated assessments

## Abstract

**Background:**

Effective debriefings in simulation-based education require accurate observation of team interactions, yet facilitators face challenges due to cognitive load, observer bias, and the complexity of team dynamics. Generative artificial intelligence (AI) tools offer a potential means to support this process by analyzing verbal communication and providing structured feedback. This study explored how AI tools can contribute to teamwork observation and debriefing in immersive medical simulations.

**Methods:**

We conducted a qualitative, exploratory study using thematic analysis of simulation participants’ and debriefers’ experiences with AI-generated teamwork reports. Forty-one participants (anesthesia nurses, residents, and attendings) participated in immersive scenarios at the University Hospital Zurich simulation center. Verbal interactions were transcribed with AI-assisted speech recognition and analyzed using two large language model–based systems (Isaac and ChatGPT-4o) guided by a prompt based on the Team-FIRST framework. Structured reports were generated for each scenario and reviewed by four simulation experts. Semi-structured interviews captured learners’ perspectives on being observed by AI tools.

**Results:**

A total of 26 AI-generated reports and 27 learner interviews were analyzed. Experts valued the detailed transcripts and illustrative quotes, which supported structured feedback and captured observations that might otherwise be missed. Limitations included inaccuracies in categorization, misattribution of speakers, overly generalized interpretations, and the absence of contextual or nonverbal information. Learners expressed openness and optimism about AI’s potential benefits: efficiency, objectivity, and enhanced perception, while also raising concerns about transparency, data protection, interpretation errors, and risks of overreliance. Both groups emphasized the necessity of human oversight.

**Conclusion:**

Generative AI tools can complement simulation debriefings by structuring communication data and highlighting teamwork patterns, supporting reflective practice. Current limitations highlight the need for multimodal approaches, refined prompting strategies, and integration with expert facilitation to ensure AI functions as a support tool rather than a replacement in simulation-based education.

**Trial registration:**

BASEC ID: Req-2024-01642.

**Supplementary Information:**

The online version contains supplementary material available at 10.1186/s41077-026-00407-0.

## Introduction

Effective team interaction is crucial for patient safety and quality of care [1], with much recent scientific emphasis being placed on defining what constitutes good teamwork in healthcare [2–4], particularly regarding psychological safety, speaking up, team learning, and reflective practice [5–8]. Simulation training has become a key method for enhancing these interactions by providing a safe environment in which to practice and refine skills [9–12]. Observing team interactions during simulation training allows training facilitators to identify strengths and weaknesses [13, 14]. This information is critical for providing feedback and facilitating reflective discussion [15–20], which are considered the core learning elements of simulation training [18, 19, 21–25]. By observing simulated interactions, it is possible to identify both helpful and unhelpful teamwork patterns [26, 27]. Targeted observation allows facilitators to guide these debriefing sessions that reinforce learning objectives [28]. However, observing team interactions presents significant challenges. First, team dynamics are neither linear nor straightforward [29–32]. Accurately perceiving these dynamics can be mentally demanding, especially in immersive simulations [33–35]. Second, observers bring their biases and preconceptions to the observation process, affecting their interpretations [36]. Third, facilitators experience significant cognitive load [37–40]. This can lead to incomplete or inaccurate observations [41]. Currently, this process is often supported by technology through integrated sound and video recording and replay systems; however, no theory-based, automated solution exists for assessing teamwork. Integrating artificial intelligence (AI) technology presents a compelling solution addressing the above challenges in simulation. Generative AI is used to support documentation and clinical decision-making [42, 43]. A subcategory of generative AI, large language models, can understand context and generate coherent, human-like language outputs [44–47]. Researchers have begun investigating AI tools, highlighting both opportunities and limitations in simulation-based education [48–52].

### Objectives

This exploratory study aimed to investigate how generative AI tools can be applied to enhance the observation of team interactions during simulation training. The AI analyzed transcripts of simulated cases and generated automated reports designed to support facilitators during post-scenario debriefing. We were interested in how experienced simulation experts assessed the quality and relevance of AI-generated teamwork reports, as well as how learners experienced being analyzed by AI tools.

## Methods

### Study design and setting

**Fig. 1:**
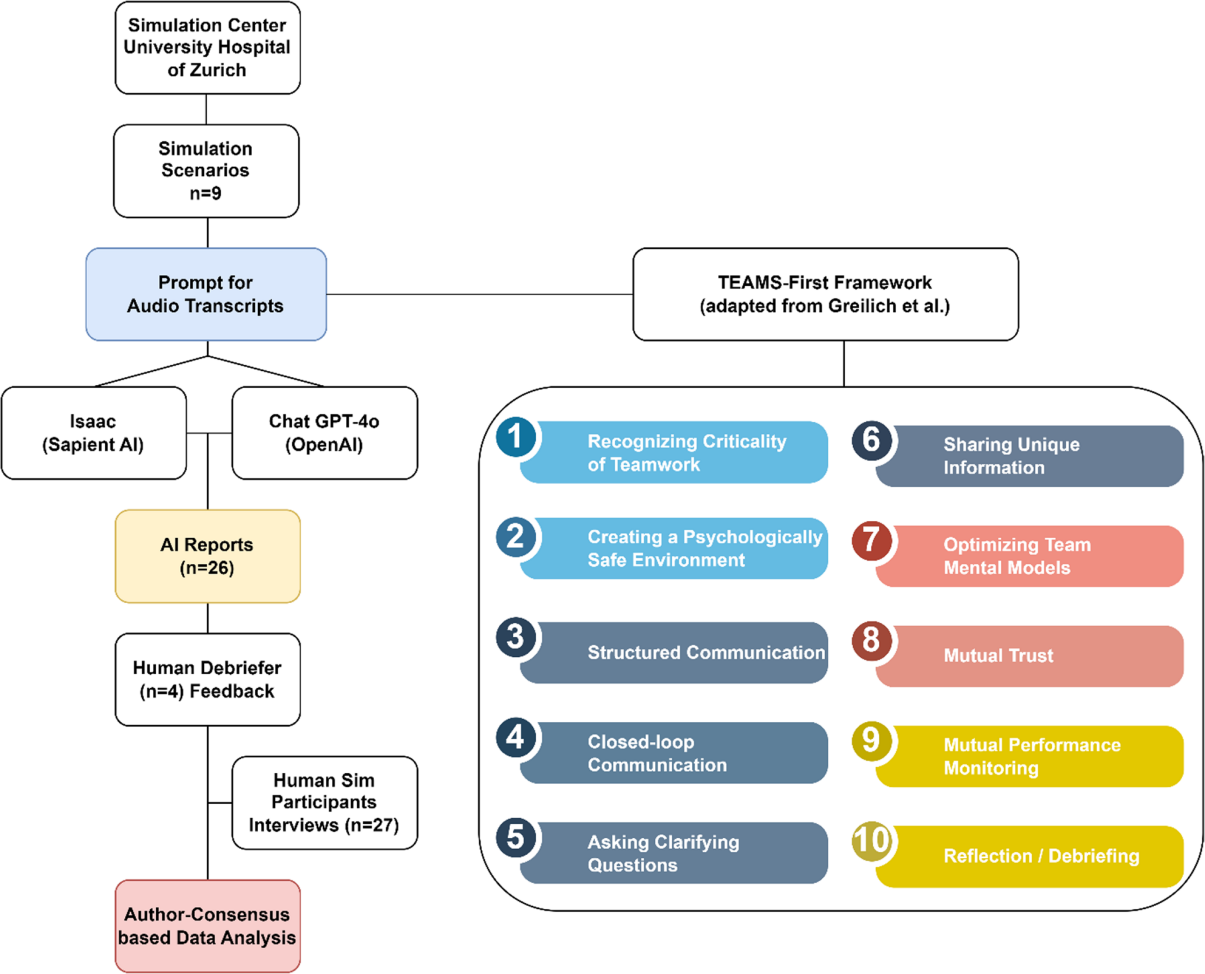
Flowchart of Study Design and Overview of the 10 Categories of the Team FIRST Framework (based on Greilich et al., 2023).

**Fig. 2:**
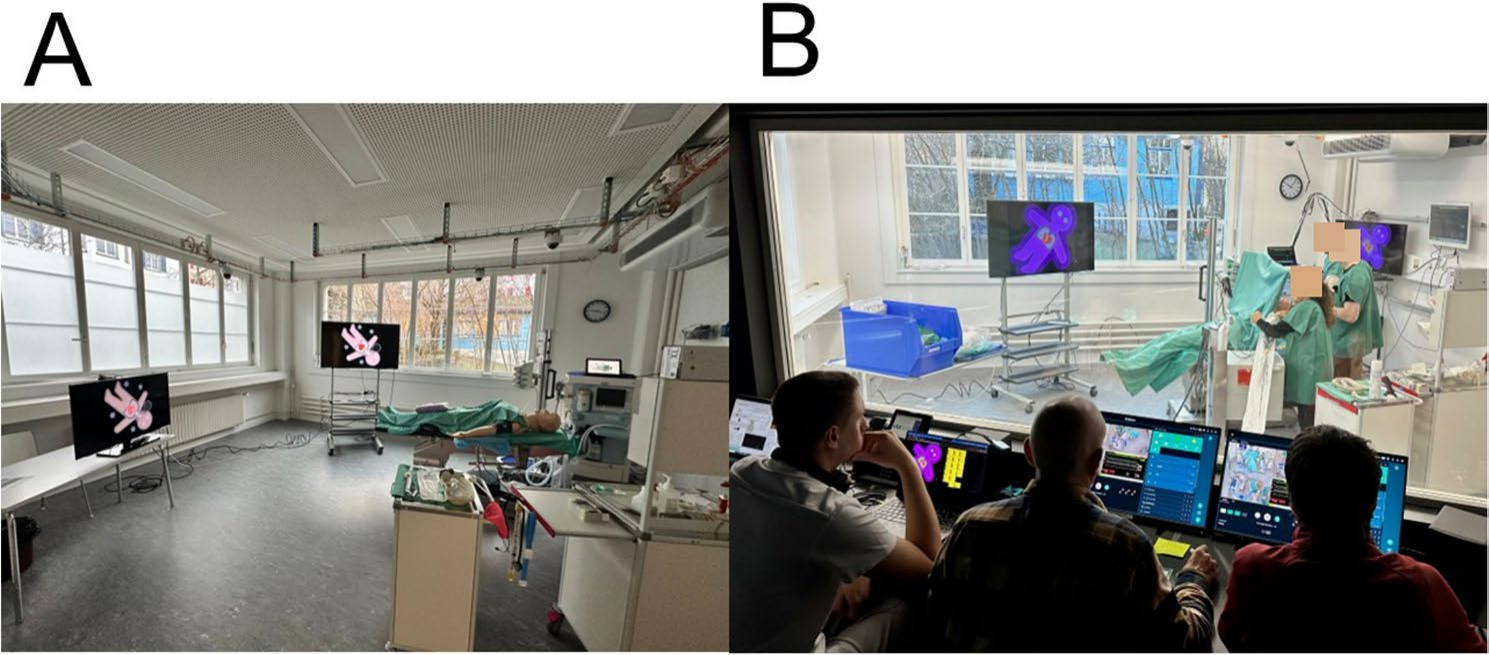
Photographs from the simulation. **A** Simulation environment at the University Hospital Zürich. **B** View from the control room, with three experts supervising a simulation scenario

In this qualitative, exploratory study, we used thematic analysis of (a) structured feedback and semi-structured interviews to evaluate simulation experts’ perceptions of AI-generated simulation reports and (b) participants’ experiences with being observed by AI tools during simulated clinical scenarios. See Fig. 1 for the study´s flowchart. We surveyed the annual, three-week simulation training program of the University Hospital Zurich’s Institute of Anesthesiology and Perioperative Medicine (January and February 2025) at the hospital’s simulation center, a state-of-the-art facility for simulation training, as shown in Fig. 2.

### Participants

Participants (total = 41), including nurses (*n* = 16), resident physicians (*n* = 17), and attending physicians (*n* = 8), were employed at the Institute of Anesthesiology and Perioperative Medicine. No specific limit or threshold on participation was set. Learners were provided with the study introduction and an explanation of our research objectives, along with an anonymized data usage agreement. No identifiable information (including names, timestamps, or participant IDs) was collected, and all data were fully anonymized. Learners did not have the opportunity to view the AI-generated reports at any point; handling of all data and reports was conducted exclusively by the study authors. See Table 1 for further Demographics.

**Table 1:** Demographics and professional background of study participants (*n* = 41)

Category	(total *n* = 41)
Gender (female)	59%
Age (years)	34 (30-43)
Experience in anesthesia (years)	7 (3-19)
Role (count)	Nurse (non-anesthesia) = 5
	Anesthesia nurse = 11
	Anesthesia resident = 17
	Anesthesia attending = 8
Experience at USZ (years)	2 (1-7)

### Simulation-based training

Simulation-based training took place during work hours, and participants received in-house Continuing Medical Education (CME) credits. Participation was voluntary. Five to seven participants trained together for a full day. Qualified clinical simulation educators led the simulation-based training. They welcomed participants and spent approximately 1–1.5 h establishing a supportive learning environment, clarifying the learning objectives, and providing orientation to the simulation equipment [53]. Subsequently, learners alternated participating in and observing their colleagues participating in two simulated cases using SimMan3G (Laerdal, Stavanger, Norway) [54]. A structured methodology was employed to develop the scenarios [55], which focused on sudden airway collapse after sedation (Scenario 1) and difficult airway management (Scenario 2). See Supplementary Table 1 for the complete simulation scripts. Scenarios were audio- and video-recorded and were used during subsequent debriefings. Two facilitators conducted the debriefings immediately after the simulated cases were complete. Debriefings focused on Team FIRST competencies and were conducted with adherence to the Debriefing with Good Judgment and TeamGAINS approaches [17, 20].

### RTF prompt design

Based on the Team-FIRST framework [3], we developed a Role Task Format prompt (RTF) to automatically generate AI debriefing reports. Structured prompt engineering has been shown to improve task specificity, clarity, and reproducibility when interacting with LLMs. It optimizes the model’s behavior toward defined tasks and reduces ambiguity in responses, which is critical for the reproducibility of LLM-prompt analysis [55]. Structured (task) inputs have also been found to improve output quality for academic uses [56]. The design of the prompt followed a clear input-to-output logic. The prompt guided the AI tools to process this material by mapping observed behaviors and communication patterns onto the ten categories defined by the Team-FIRST framework. The expected output was a report in which each category was presented as a separate section containing a summary and examples extracted from the transcript. Figure 1 illustrates the ten categories of the Team-FIRST framework. See Supplementary Fig. 1 for the complete RTF prompt.

### Integration of AI tools for team verbal communication transcription and teamwork analysis

We asked the participants to wear RØDE lapel microphones while participating in the simulated cases, with audio signals transmitted directly to an iPad running the Isaac software (Saipient AI, Switzerland). This system generated real-time automatic transcripts of verbal team communication. We uploaded these transcripts into two separate AI-based tools (with the same underlying large language model) for analysis: (a) Isaac by Saipient AI and (b) ChatGPT-4o by OpenAI. Both then compiled a separate and annotated report (Supplementary Material). Transcripts were uploaded immediately after scenario completion. The AI tool analysis took less than two minutes. Learners did not receive copies of the AI-generated reports. Transcripts were generally accurate and clear according to the simulation experts (and the study’s authors), allowing reliable analysis. Minor errors occurred in speaker identification, particularly for statements delivered by the overseeing facilitators when giving external cues and commands using the voice-over loudspeaker (Audio to Room).

### The debriefers’ perspective: evaluation of AI-generated teamwork reports

Four members of our study group (JCS, MK, HH, MT), experienced simulation experts, evaluated the AI-generated reports. Author DWT assigned them two reports per scenario: one report for each AI tool. The origin of the report was blinded to the debriefers. The debriefers were asked to comment on the selection, structure, interpretation, and relevance of the content, as well as share any emotional reactions by open-ended written commentary (i.e., instruction:*“My thoughts on the report (report begins below the line): Please enter your thoughts and feelings here.”*).

### The learners’ perspective: evaluation of learners’ reactions

To understand how learners experienced being analyzed by AI tools, authors DWT, AR, and HW interviewed them after they completed their scenarios at the end of the training day. The guiding question was:

*“*What are your thoughts and feelings about being observed by AI during the simulation? Please share anything that comes to mind.*”*

### Data analysis

We analyzed the learners’ written interview responses and the evaluators’ comments using a thematic analysis approach [57]. DWT and AR reviewed all raw responses and developed initial codes. They iteratively structured and consolidated the data into overarching thematic clusters, using Microsoft Word, with each response assigned a corresponding code. The resulting coding structure was reviewed, discussed, and validated with co-authors JCS and MK. The resulting categories and sample quotes from debriefers’ evaluations of the AI-generated teamwork reports are presented in Table 2. The categories and sample quotes from learners’ reactions to being analysed by AI tools are shown in Table 3.

**Table 2:** Categories and example quotes from debriefers. The table presents key themes, subthemes, and illustrative comments from simulation debriefers on the quality and usefulness of AI-generated reports

Theme	Subtheme	Examples
**Positive Feedback**	Broader Observation Capture	*“Examples [quotes] are appropriate. (However*,* I don’t remember them — which doesn’t necessarily mean anything*,* as there was a lot of noise in the control room and unrest in the scenario.)”*
	Quote Selection Supports the Theme Well	*“I share the above assessment — from my perspective*,* it’s perfect.”*
	Support for Debriefing	*“I would benefit from this transcript in a debriefing.”*
**Categorization Accuracy Issues**	Unclear, Inaccurate, or Overlapping Categorization	*“The psychologically safe environment was present — but I didn’t see it reflected in these quotes.”*
	Overgeneralized, Superficial, or Ambiguous Categorization	*“These two points from the “mutual trust” category seem strongly out of context to me.”*
	More Representative Quotes Could Have Been Chosen	*“For me*,* structured communication would mean something like an ABCDE or a 10-for-10; the above statements are*,* in my opinion*,* not examples of that. I heard and coded an ABCDE during the scenario*,* and its content was different.”*
	Desire for More Context or Stronger Exemplification	*“I think the statements are part of structured communication; what was said before or after? Additional information would help me form an opinion.”*
**Identification of Problems**	Misidentification of Speaker or Role	*“I believe this information also came from outside the team and perhaps shouldn’t be included here. We would need a way to mark speakers and exclude certain roles from interpretation — for example*,* instructors or embedded simulated persons (ESPs).”*
	Misinterpretation of Technical Terms or Abbreviations	*“There are issues with the handling of technical vocabulary*,* although the key aspects were still recognizable.”*
	Overconfident or Unwarranted Wording by AI	*“Strange conclusion — how did the AI come up with that?”*
	Systematic Biases in AI Interpretation	*“It would also be interesting if central aspects that were missing could be suggested — for example*,* lack of coordination*,* absence of closed-loop communication*,* or the use of destructive language.”*
**Suggestions for Improvement**	Link Quotes to Timestamps and Identify Speakers	*“What I’m missing here are the names of the speakers. I find the timestamps extremely helpful for understanding the context and potentially showing the corresponding scene in the video.”*
	Expand Beyond Text-Based Analysis	*“I could also imagine that the AI might be able to distinguish the participants’ speaking volume over time — for example*,* when someone speaks loudly or quietly*,* and what impact that has on the team.”*
	Improve Result Presentation and Clarity	*“The quotes could be shown in a list and color-coded by theme—whether the AI classified them as relating to challenges*,* communication*,* or coordination skills.”*

**Table 3:** Categories and example quotes from participants. The table shows selected categories and corresponding illustrative statements reflecting participant perspectives on AI-based observation and perceived benefits

Theme	Subtheme	Examples
**Influence of perceived observation**	-	*“The fact that AI is listening and creating a transcript doesn’t change how I feel.”*
**Perceived Benefits**	General Optimism About the Potential of AI Technologies	*“AI is the future*,* definitely. You have to play along*,* or you’ll miss the trend. It’s a race — if you miss the jump*,* you’re left behind.”*
	Trust in AI	*“Not really a problem with how it’s being used.”*
	Enhanced Perception Through AI	*“An additional aid to humans*,* because it notices more.”*
	Support for Generating Ideas and Structuring Feedback	*“It can provide interesting input and be helpful when things are being compared.”*
	Increased Efficiency Through Automation	*“It can help us summarize information.”*
	Objectivity and Impartiality	*“Computers take emotions out of the equation — that might make them fairer in terms of evaluation.”*
	Independence from Human Factors	*“A human can’t be completely objective; AI is objective. AI looks only at what is given and puts it down on paper. That can be an advantage and a disadvantage.”*
**Perceived Risks**	Lack of Transparency	*“You’re left in the dark about the background — it gives you an uneasy feeling.”*
	Data Protection and Security Concerns	*“Conflicted about data protection: Where does the data go? How is it processed?”*
	Interpretation Errors	*“There may be a lack of understanding. AI certainly can’t empathize with people as well — humans are better at that.”*
	Loss of Cognitive, Communicative, and Social Abilities	*“Fear of neglecting one’s own intuition.”*
	Lack of Trust in AI	*“The lack of emotion is also a disadvantage*,* because certain aspects go unnoticed.”*
	Various Concerns About AI Technology	*“Can AI understand all dialects*,* all languages?”*
**Suggested Key-Features for AI**	Support for Human Situation Awareness	*“I want feedback to know that the AI understood what it was for.”*
	Desire for Human Involvement in Evaluation	*“The interpretation and debriefing should be led by a human.”*

### Reflexivity

As authors of this manuscript, we come from medicine, nursing, and psychology and represent a range of career stages, with shared experience in designing, delivering, and studying simulation-based education. Our backgrounds and professional roles shaped both our perspectives and our analytic focus. DWT, BG, and MK had prior experience with the use of AI in developing novel visual patient monitoring systems [58, 59], whereas AK, MT, MK, JCS, and BG had substantial expertise in team interaction analysis [36, 60–63] and interprofessional simulation training [15, 20, 26, 64]. Throughout the study, ongoing interdisciplinary discussions led us to refine our assumptions, particularly by integrating psychological and nursing perspectives into the interpretation of observed effects.

## Results

A total of 26 AI-generated reports from nine different simulation scenarios, conducted between January 21 st and January 28th 2025, were reviewed and commented on by four simulation experts. Additionally, 27 participants participated in interviews about their experiences with AI-based observation and analysis during the simulations; see Table 1 for the demographics of the simulation participants.

### The simulation experts’ perspective on AI

We have identified four different themes in simulation experts’ perspectives on AI-generated teamwork reports: (1) positive feedback, (2) categorization accuracy issues, (3) identification of problems, and (4) suggestions for improvement. Table 2 provides selected quotes from the simulation experts’ comments. The coding trees derived from these debriefers’ responses are presented in Supplementary Fig. 2. Supplementary Table 2 provides the full feedback from the simulation experts. While we did not specifically ask whether the AI tools’ output would influence the simulation experts’ personal practice, several commented that the report could serve as a helpful adjunct during debriefings.

### Simulation experts’ theme 1: positive feedback

“*Broader Observation Capture*” emphasized that AI-generated transcripts often captured details that simulation experts missed during live observation. “*Quote Selection Supports Theme Well*,” captured views that the generative AI tools selected quotes that effectively illustrated specific and relevant aspects of teamwork: “*A participant stated that the (AI tools) remarks helped to build trust*,* spontaneously and without the topic having been discussed (further by the debriefer).”* Lastly, “*Support for Debriefing*,” reflected debriefers’ perception that the concept offered valuable assistance for debriefing practice.

### Simulation experts’ theme 2: categorization accuracy issues

This theme relates to the difficulties AI tools face in accurately categorizing transcribed quotes into the Team FIRST categories. “*Unclear*,* Inaccurate*,* or Overlapping Assignment*” reflected that quotes did not consistently align with the competencies, while “*Overgeneralized*,* Superficial*,* or Ambiguous Categorization”* captured that classifications lacked depth, specificity, or sufficient contextual justification. Finally, the “*Desire for More Context or Stronger Exemplification*” summarized the request for longer or more complete dialogue excerpts.

### Simulation experts’ theme 3: identification of problems

The first subtheme, “*Identification of Problems: Misidentification of Speaker or Role*” captured concerns about inaccuracies in attributing statements to the correct team members. *“Identification of Problems: Misinterpretation of Technical Terms or Abbreviations*” reflected debriefers’ observations that technical language was sometimes inaccurately captured or translated. “*Overconfident or Unwarranted Wording by AI”* captured concerns that some statements lacked critical nuance. One debriefer stated: *“The wording is very assertive and explicit.”* The final subtheme, “*Systematic Biases in AI Interpretation*” captured concerns that the AI tools may have exhibited implicit assumptions in their interpretation of team dynamics. One debriefer questioned, *“I wonder what knowledge the statement is based on: that this happened between a doctor and a nurse. Could a bias be at play*,* assuming certain roles automatically belong to certain professions?”*

### Simulation experts’ theme 4: suggestions for improvement

The first subtheme was *“Link Quotes to Timestamps and Identify Speakers”.* The second subtheme, “*Expand Beyond Text-Based Analysis*” highlighted concerns that important nonverbal cues, such as tone of voice, body orientation, and overall atmosphere, were not captured. One remarked, *“The calm and controlled tone that contributed to psychological safety is missing. This is something we pay close attention to as instructors.”* The third subtheme was “*Improve Result Presentation and Clarity”*. One suggestion was to embed timestamped quotes directly into existing tools.

### The learners’ perspective on AI

Based on the interviews with learners, we have identified four different themes in their perspectives on being observed and analyzed by an AI tool during simulation-based training: (1) influence of perceived AI observation, (2) perceived benefits, (3) perceived risks, and (4) suggested key features for AI. Table 3 presents corresponding examples from the participant interviews. Supplementary Fig. 3 and Supplementary Table 3 provide the full feedback from learners.

### Learners’ theme 1: Influence of Perceived AI Observation

This theme encompassed participants’ varying reactions to being aware of being observed by AI tools. For some learners, the presence of AI tools faded into the background and did not influence their behavior. Others described a general sense of inhibition, regardless of who or what was watching:

*“You don’t feel entirely free to act. Just the fact of being watched causes tension*,* whether it’s AI or a human.”*

### Learners’ theme 2: perceived benefits

The first subtheme, “*General Optimism About the Potential of AI Technologies*”, captured participants’ hopeful outlook on the role of AI tools in clinical settings. The second subtheme, *“Trust in AI”*, included participants’ statements expressing high confidence in AI tools and little concern about potential risks. ”*Enhanced Perception Through AI”* included learners’ reflections, as one remarked, *“As humans*,* we can only perceive so much. […] AI might help us notice other things.”* The fourth subtheme, *“Support for Generating Ideas and Structuring Feedback”*, included participants’ views of AI as a valuable partner in creative and analytical processes. *“Increased Efficiency Through Automation”* and *“Objectivity and Impartiality” captured the notion that: “Unlike human analysis*,* it prevents bias based on past mistakes.”* The final subtheme, *“Independence from Human Factors”*, included participants’ appreciation of AI’s ability to operate without being directly influenced by human biases.

### Learners’ theme 3: perceived risks

The first subtheme, *”Lack of Transparency”*, included participants’ concerns: *“AI can present facts in a way that makes them seem true. With humans*,* you notice uncertainty or emotion. A computer appears more confident. But is the information correct?”* The second subtheme, *“Data Protection and Security Concerns”*, included participants’ unease about how their data is stored, processed, and shared. *“Interpretation Errors”* included participants’ concerns about the risk of AI misinterpreting input. *“Loss of Cognitive*,* Social*,* and Communicative Abilities” and “Lack of Trust in AI” included participants’ concerns about the long-term impact of AI tools* on essential human skills. One remarked, “*You don’t need communication skills anymore*,” referring to how AI tools increasingly take over tasks like writing or speaking. *“Various Concerns About AI Technology”* included a broad range of practical and ethical questions raised by participants.

### Learners’ theme 4: suggested key features for AI

*Support for Human Situation Awareness” included participants’ reflections on how AI tools’* output should be accessible and applicable. They emphasized that *“being easy to perceive will be the key. It must be output that people notice.”* “*Desire for Human Involvement in Evaluation” included participants’ insistence on needing expert oversight when using AI tools*. One noted that *“AI is only as good as the input you give it*,*”* and its output must be *“evaluated with expert knowledge.*

## Discussion

In this exploratory study, we examined the current capabilities of two AI tools to generate teamwork reports from transcripts of simulated cases, with the aim of supporting post-scenario debriefing rather than conducting a comparative evaluation. Experienced simulation experts evaluated the quality and relevance of the AI-generated reports, while learners reflected on their experiences of being observed and analyzed by AI tools. Simulation experts viewed the reports as valuable adjuncts for debriefing due to their ability to capture overlooked interactional details and provide structured illustrative quotes, while also identifying limitations related to categorization accuracy, contextual understanding, and potential bias. Learners expressed general optimism about the AI tool’s potential benefits, including efficiency and perceived objectivity, alongside concerns about transparency, data protection, and the impact on communication skills.

### Simulation experts’ experiences with AI-generated teamwork reports

A central strength of AI-generated reports was what experts described as “*broader observation capture*”. Both AI tools offered relevant interactional details that would have likely escaped the expert’s notice during the scenario. This observation is consistent with constraints of real-time observation in immersive simulation [38, 64]. Scenario coordination, alongside attentive analysis of team dynamics, does occur in a linear or clearly demarcated sequence; instead, it merges through rapid, overlapping verbal and nonverbal exchanges. Even experienced facilitators, operating under significant cognitive load, may miss subtle but important moments in interactional dynamics [31, 65–67]. In this context, AI-generated transcripts and selected quotes can serve as a post-hoc reconstruction, enabling facilitators to revisit sequences with greater granularity. The AI tools showed to have the ability to capture illustrative quotes that aligned with teamwork dynamics, with one simulation expert noting that: “*the [AI tools] remarks helped to build trust*,* spontaneously and without the topic having been discussed [further by the debriefer].”* This aligns with emerging evidence that AI tools can produce clinically relevant summaries with reasonable completeness when guided by structured prompts, even though contextual fidelity remains a challenge [68, 69]. This was closely tied to concerns related to the unclear, inaccurate, or overlapping assignment of quotes and categorizations, as well as insufficient contextual justification. Categorizing teamwork behaviors is not a purely lexical task; it requires sensitivity to intent, timing, role expectations, and sequential dependencies. These concerns relate to critiques of AI tools in healthcare, where hallucinations and context-insensitive outputs pose risks [70]. Simulation experts did not reject the AI tools categorization in many cases, but emphasized the need for richer contextual embedding, such as longer dialogue excerpts. A major concern raised by simulation experts is the misidentification of speakers and what was perceived as overconfident or unwarranted wording. Experts also questioned whether AI implicitly inferred professional roles based on speech patterns or content, for example, if a directive statement originated from a physician rather than a nurse. One debriefer explicitly asked whether “*a bias [could] be at play*,* assuming certain roles automatically belong to certain professions?*” This observation aligns with the broader literature on bias in AI tools, which reflects the sociocultural patterns embedded in training data and may perpetuate hierarchical assumptions unless explicitly addressed [71]. Human observers also bring expectations, schemas, and role stereotypes to their evaluations. However, whereas human biases are often reflected upon and distributed across multiple facilitators, AI biases may be systematic and replicated on a large scale [72, 73]. Simulation Experts suggested practical mitigation strategies, including manual speaker identification as input to the AI tool, linking quotes directly to timestamps, and pairing AI outputs with written context prior to analysis. Human validation of AI-generated reports could create a bias-checking loop, ensuring that no single interpretive source, human or AI tool, dominates the debriefing narrative. Without workflow optimization, AI tools risk shifting rather than reducing cognitive burden, forcing facilitators to expend additional effort reconstructing who said what, when, and in response to which event. This concern aligns with findings from prior work, which indicate that the introduction of AI into debriefing can increase facilitators’ mental demand if systems are not streamlined and well-integrated [50]. From a systems perspective, these observations reinforce the importance of human-in-the-loop (HITL) design. AI tools are increasingly capable of autonomously generating structured reports, but the literature consistently emphasizes that they do not replace the need for human judgment, contextual understanding, and accountability [74]. Effective HITL systems actively integrate human expertise into the AI decision-making cycle, rather than relegating it to post-hoc review [75, 76]. Evidence from clinical contexts suggests that expert oversight of AI-generated text enhances quality and reduces risk compared to unattended automation, albeit with trade-offs related to efficiency and cognitive load [77]. In simulation debriefing, where interpretive nuance and pedagogical intent are central, such trade-offs may be both inevitable and acceptable. A further limitation identified by simulation experts was the absence of nonverbal information in AI-generated reports. While text-based transcripts capture what was said, they fail to represent how it was said and how it was embodied in its situational, simulated context. Experts have noted that critical teamwork constructs are often conveyed through tone of voice, prosody, body orientation, gaze, and overall atmosphere. One simulation expert remarked that *“the calm and controlled tone that contributed to psychological safety is missing.”* This limitation is known to affect teamwork research, where nonverbal communication is recognized as a crucial component of coordination and trust [7, 64]. From a technical standpoint, integrating audio and video into live AI tool analysis is feasible, offering richer data for interpretation. Combining audio, text, and video can enhance analytical depth [78]. However, practical constraints, including the need for high-resolution recordings, data upload, and governance challenges, as well as the ethical implications of streaming sensitive audiovisual data, exist, making it difficult for simulation centers to live-upload video into AI tools. Text-based systems may be enhanced through improved contextualization, speaker identification, and integration with scenario timelines [79]. Multimodal systems incorporating selected audio or video segments may offer more faithful representations of team dynamics, provided that governance frameworks and human oversight remain central to their operation [80].

### Learners’ experiences of being observed and analyzed by AI

Learners’ perspectives on AI-based observation revealed optimism tempered by caution. Some learners reported that the presence of AI tools faded into the background and did not influence their behavior, while others described a sense of inhibition associated with being observed, regardless of whether the observer was human or artificial. This suggests that the mere awareness of observation, rather than the AI tools per se, may shape learner behavior, a phenomenon well described in educational and organizational psychology [81]. Many learners expressed enthusiasm about the potential benefits of AI. They envisioned AI as a tool that could enhance perception, structure feedback, and increase efficiency by automating aspects of analysis. Many emphasized objectivity and impartiality, contrasting AI with human observers who may carry biases or be influenced by prior experiences. This perceived independence from human factors aligns with arguments in favor of technical performance assessment in complex team settings, where subjective interpretation can compromise reliability [64]. At the same time, a “*Lack of transparency*” emerged as a central concern. Participants questioned how AI-generated outputs were produced and whether they could be trusted, particularly given the confident tone often adopted by AI systems. One learner noted that “*AI can present facts in a way that makes them seem true*,*”* highlighting the risk that errors may go unnoticed precisely because AI outputs lack the emotional cues and uncertainty that characterize human feedback. These concerns mirror those raised by simulation experts and align with broader debates about explainability and trust in AI systems used in healthcare [82]. Learners expressed unease about how their data were stored, processed, and potentially shared. Concerns about misinterpretation extended to linguistic issues, including local dialects and abbreviations. Together, these observations highlight the importance of transparent communication regarding data handling, technical limitations, and error mitigation strategies. Learners also reflected on potential long-term risks associated with increasing reliance on AI tools. Some worried about the erosion of cognitive, social, and communicative skills if AI tools were to assume roles traditionally fulfilled by human interaction [83]. Learners strongly emphasized the importance of human involvement in interpretation and decision-making, and this reflects foundational principles of human-centered AI design [84]: systems should enhance, not replace, expert judgment; AI-generated output should be transparent, interpretable, and actionable; and they should respect the social and ethical dimensions of educational practice.

### Limitations and future research

This study was conducted at a single simulation center, which had a limited number of scenarios and simulation experts. The results provide qualitative depth but are not generalizable. Moreover, the analysis relied exclusively on one RTF prompt and verbatim transcripts, which included local dialects, potentially introducing transcription-related errors and limiting the AI’s interpretive accuracy. The AI tools were not trained or fine-tuned on domain-specific simulation data. Supervised HITL may improve output reliability, as future research could explore a live-feed multimedia approach that combines video, audio, and context through annotations from human facilitators directly to AI tools. The authors’ prior experience inevitably influenced study design and interpretation. All authors have professional expertise in simulation-based education and qualitative methods, including thematic analysis. We recognize that our perspectives may have shaped data interpretation, coding decisions, and the framing of themes. To mitigate potential bias, multiple researchers independently reviewed transcripts and AI-generated outputs, and discrepancies were resolved through discussion. Although participants did not have access to AI-generated debriefings, they did not perceive AI as threatening, while expressing concerns regarding privacy, data storage, and AI-derived analyses. Consistent with recent interview studies, participants appear receptive to AI-generated feedback when it provides concrete, relevant suggestions, is embedded within a guided feedback process, and is not used as a standalone evaluative tool [85]. AI-generated reports may serve as a standardized baseline to identify recurring themes, communication dynamics, or technical skills of participants, and should help open the facilitator-led discussion. Future research should focus on integrating AI tools with live, multimodal inputs, including video, audio, and HITL context, to support debriefing in complex simulation environments.

## Conclusion

AI tools, such as ChatGPT and Isaac, can provide a structured report in simulation-based education by organizing team communication and highlighting relevant interactions. These may help facilitators and learners rapidly identify key events, recurrent themes, and potential learning objectives, thereby improving efficiency and focus on the outset of the debriefing. Current limitations in contextual understanding and nuanced interpretation underscore the need for these systems to complement, rather than replace, human-led debriefing, as it is dependent on interactive discussion.

## Supplementary Information

Supplementary Material 1

Supplementary Material 2

## Data Availability

Most data generated or analysed during this study are included in this published article (see Supplementary Tables 1 and Supplementary Table 2).
