# Supplementary material for: Generative AI in simulation debriefings: an exploratory study using the Team-FIRST framework and qualitative feedback from simulation experts and learners
Source: Adv Simul (Lond). 2026 Jan 29;11:14. doi: 10.1186/s41077-026-00407-0 (PMC12924402; doi:10.1186/s41077-026-00407-0)
Supplement: Supplementary file 1 — Supplementary Material 1 [file 41077_2026_407_MOESM1_ESM.docx]

Supplementary Information to  **Generative AI in Simulation Debriefings: An Exploratory Study Using the Team-FIRST Framework**

David W. Tscholl^+^, Max Ebensperger^+^, Arend Rahrisch, Helius Wang, Hubert Heckel, Max Thomasius, Alexander Kaserer, Bastian Grande, Julia C. Seelandt^*^, Michaela Kolbe^*^

+, * contributed equally

**Corresponding author:** Michaela Kolbe

**Email:** michaela.kolbe@usz.ch

**Role:** You are a simulation expert analyzing a transcript recorded during realistic medical simulations. The transcript documents team communication.

**Task:** Please analyze the transcript provided and create a structured, in-depth report that analyzes the following 10 teamwork competencies. Your report should assist simulation experts in conducting debriefings with the team. Use complete and lengthy quotes from the transcript to support your analysis, embedding them meaningfully. Consider communication within the overall context and anonymize any names automatically.

1. **Recognizing the Importance of Teamwork**
   Was the central role of teamwork recognized and emphasized? Were different interprofessional roles understood and valued? Highlight exceptional or contrasting examples.
2. **Creating a Psychologically Safe Environment**
   Are there signs that team members can freely express themselves, ask questions, seek help, or admit mistakes? Provide 3 examples.
3. **Structured Communication**
   Were clear and structured communication protocols or common terminologies used? Provide 3 examples.
4. **Closed-Loop Communication**
   Were messages received, acknowledged, and acted upon correctly? Provide 3 examples.
5. **Asking Clarifying Questions**
   Were clarifying questions used to share knowledge or avoid misunderstandings? Provide 3 examples.
6. **Sharing Unique Information**
   Were critical pieces of information shared that other team members may not have had? Provide 3 examples.
7. **Optimizing Shared Mental Models**
   Are there indications of a shared understanding within the team regarding patient history, priorities, emergency planning, or responsibilities? Provide 3 examples.
8. **Building Mutual Trust**
   Is it evident that team members build and maintain mutual trust? Provide 3 examples.
9. **Mutual Performance Monitoring**
   Did team members monitor and support each other to ensure high-quality care? Was feedback given and accepted constructively? Provide 3 examples.
10. **Reflection and Debriefing**
    Are there indications of reflection on current experiences or structured debriefings to learn from the simulation? Provide 3 examples.

- The report should be well-structured and easily readable.
- Use subheadings for each of the 10 points of analysis.
- Limit the report to two A4 pages.
- Analyze communication within the overall context rather than in isolation.

**Supplementary Figure S1** Prompt provided to both AI programs.

| **Scenario 1:** | | | |
| --- | --- | --- | --- |
| For participants | For instructors | Input | Checklists |
| **Participants** | | | |
| All participants have to wear the standard-issued OR clothing without facemasks.  We are in a PACU setting. | | | |
| Immediately available personnel | | On-call | |
| PACU nurse | | Anesthesia nurse  Anesthesia resident  Anesthesia attending | |
| **Scenario script for participants** | | | |
| We are in the PACU N2C. It is just before 7 a.m. As sometimes happens, Mrs. Tobler will be brought to the PACU preoperatively in a few moments, as she is an inpatient in our surgical centre. She was born in 1967 and is scheduled for elective surgery for a tongue-base carcinoma. Height 163 cm, weight 42 kg. The electronic anaesthesia record (eBA) includes, among other things, the following information: awake fibreoptic nasal intubation, postoperative tracheotomy, and recovery in the PACU. | | | |
| **Briefing for participants** | | | |
| Situation in N2C OR at the start of the workday. | | | |
| The ward nurse (together with the transport service) is standing by the bed in front of the PACU door. The patient is wheeled to the PACU bay. Handover from ward to PACU: “*Mrs. Tobler, as discussed yesterday, is coming to the PACU preoperatively. She couldn’t sleep and requested something to calm her down; after consulting with the ward physician, she received 7.5 mg Dormicum at 5 a.m.”* (If asked further questions by the team: “*Oh, I don’t know — I was just told to bring her over*.”) | | | |

| **Scenario script for Sim-Staff** | |
| --- | --- |
| **Material for Simulation** | |
| Dummy in patient gown No lines or devices, no monitoring Unresponsive (GCS 8 with 2-2-4) | |
| **Starting settings** | **Diagnosis** |
| Eyes closed SpO₂ 85% Bradypnea Cyanotic lips  Bilateral normal breath sounds | No response to verbal stimuli Hypoxia Hypoventilation (Dormicum overdose)  Reduced vigilance |
| **Scenario development** | |
| Event | Expected Actions |
| \| Hypoxia \|  \| \| --- \| --- \| | Mask ventilation, antidote with flumazenil: minimal improvement |
| \| Cannot ventilate \|  \| \| --- \| --- \| | Sedation, muscle relaxation |
| \| Still cannot ventilate (CNV) \| \| --- \| | Laryngeal mask airway insertion |
| \| Still CNV \|  \| \| --- \| --- \| | Direct/indirect laryngoscopy or endoscopy |
| \| Cannot intubate \| \| --- \| | Return to laryngeal mask airway? Or cricothyrotomy |
| \| If cricothyrotomy delayed \|  \| \| --- \| --- \| | Hypoxic cardiac arrest |

| **Mindset for ESP** |
| --- |

| **Learning Objectives** | |
| --- | --- |
| **Technical** | **Behavioral** |
| - **“Where do I position myself for the cricothyrotomy?”** - **“Where and how do I make the incision?”** - **Positioning: Head end? Bed against the wall?** | **Leadership skills:** - Preparation for difficult airway situations, preemptive strategies (e.g., awake fiberoptic intubation, supraglottic airway devices) - Who performs the cricothyrotomy? - Speak up, closed-loop communication - Managing cognitive load: task distribution, task delegation, maintaining attention under stress (10-for-10 principle) |

| **Checklists** | |
| --- | --- |
| **Start position, installations** | |
|  | |
| **Start positions** | **Cleanup, debriefing** |
| **Anesthesia personnel** | |
| **Equipment to bring for in situ** |  |
| **Dummy prepared with cricothyrotomy capability**  **New difficult airway trolley** |  |
| **Operating Room (OR)** | |

| **Scenario 2:** | | | |
| --- | --- | --- | --- |
| For participants | For instructors | Input | Checklists |
| **Participants** | | | |
| Everyone in their role. Scenario starts on cue, do not make arrangements beforehand, otherwise we don’t hear them and assume wrong things Green OR gowns, no masks. We end the scenario on cue. | | | |
| Immediately available personnel | | On-call | |
| Anesthesia nurse  Anesthesia resident | | Anesthesia attending | |
| **Scenario script for participants** | | | |
| In Room 2 is Mr. Rutschli for gastric bypass. He was fiberoptically awake intubated due to an expected difficult airway. Surgery begins in a few minutes. | | | |
| **Briefing for participants** | | | |
| Situation in N2C OR at the start of the workday. | | | |
| This is Mr. Rutschli, 60 years old, 180 cm tall, 140 kg. He has mild arterial hypertension and known sleep apnea, allergy to house dust and pollen. He is here for a gastric bypass under general anesthesia. He was just fiberoptically awake intubated. The intubation went well. However, he has had high ventilation pressures from the start. He has a 17g cannula and a urinary catheter. The surgical area is disinfected and covered, and the visceral surgery attending will arrive any moment. A: intubated with 7.5 tube, tooth row 23 B: tidal volume 500, rate 16, inspiratory pressure 29, PEEP 8 C: stable, has 17g cannula D: sleeping with 0.2 fentanyl, sevoflurane, Propofol TCI 4, had 100 Rocuronium E: 200 ml urine in urinary catheter | | | |

| **Scenario script for Sim-Staff** | |
| --- | --- |
| In Room 2 is Mr. Rutschli for gastric bypass surgery. He was fiberoptically intubated. Everything went well. Ventilation pressures high from the start, circulation stable. **While positioning arms, the positioning nurse trips and accidentally pulls out the tube and cannula.**  Mannequin covered for laparoscopy. Arms positioned. A: intubated, B: ventilated, high resistance, tidal volume 300, rate 16, PEEP 8, SpO2 94, C: existing cannula connected, 17g cannula taped Infusion with red stopper and propofol running towards infusion, Pulse 88, BP 135/86, D: eyes closed, E: urinary catheter Available before surgery: Intubation trolley, C-Mac, fiberoptic, DIC trolley | |
| **Starting settings** | **Diagnosis** |
| Ventilated, rate 16, tidal volume 300, PEEP 8 Pulse 88, BP 135/86, saturation 94 | Arterial hypertension Sleep apnea Obesity |
| **Scenario development** | |
| Event | Expected Actions |
| \| Tube disconnected \|  \| \| --- \| --- \| | Saturation falls to 40 within 6 minutes |
| \| Cannula pulled out \|  \| \| --- \| --- \| | Pulse rises to 140 over 10 minutes, BP to 170/100 |
| \| Mask ventilation Laryngeal mask airway (LMA) \|  \| \| --- \| --- \|   Fiberoptic via LMA  Cricothyrotomy | Doesn’t work, high resistance Minimal ventilation possible, high resistance 10 for 10 should be done for further procedure Not adjustable, possibly red overlay Possible Saturation rises |

| **Mindset for ESP**  You are responsible for positioning. After 2 minutes the surgeon calls and asks you to position the arms out. During this you trip, and the tube and cannula are accidentally pulled out. The anesthesia team can deploy you as a positioning nurse according to your competencies. |
| --- |

| **Learning Objectives** | |
| --- | --- |
| **Technical** | **Behavioral** |
| - **Algorithm unexpected difficult airway** | Cognitive overload Task overload PP TEAM FIRST FRAMEWORK Creating psychological safety Structured communication Sharing information, speaking up Team timeout (when and how) |

| **Checklists** | |
| --- | --- |
| **Start position, installations** | |
|  | |
| **Start positions** | **Cleanup, debriefing** |
| **Anesthesia personnel** | |
| **Equipment to bring for in situ** |  |
| Dummy prepared with cricothyrotomy capability  New difficult airway trolley |  |
| **Post-training Input** Send all participants the algorithm again by email 2 weeks after the simulation. | |

**Supplementary Table S1** Illustrating Scenario Scripts for Scenario 1 and Scenario 2.


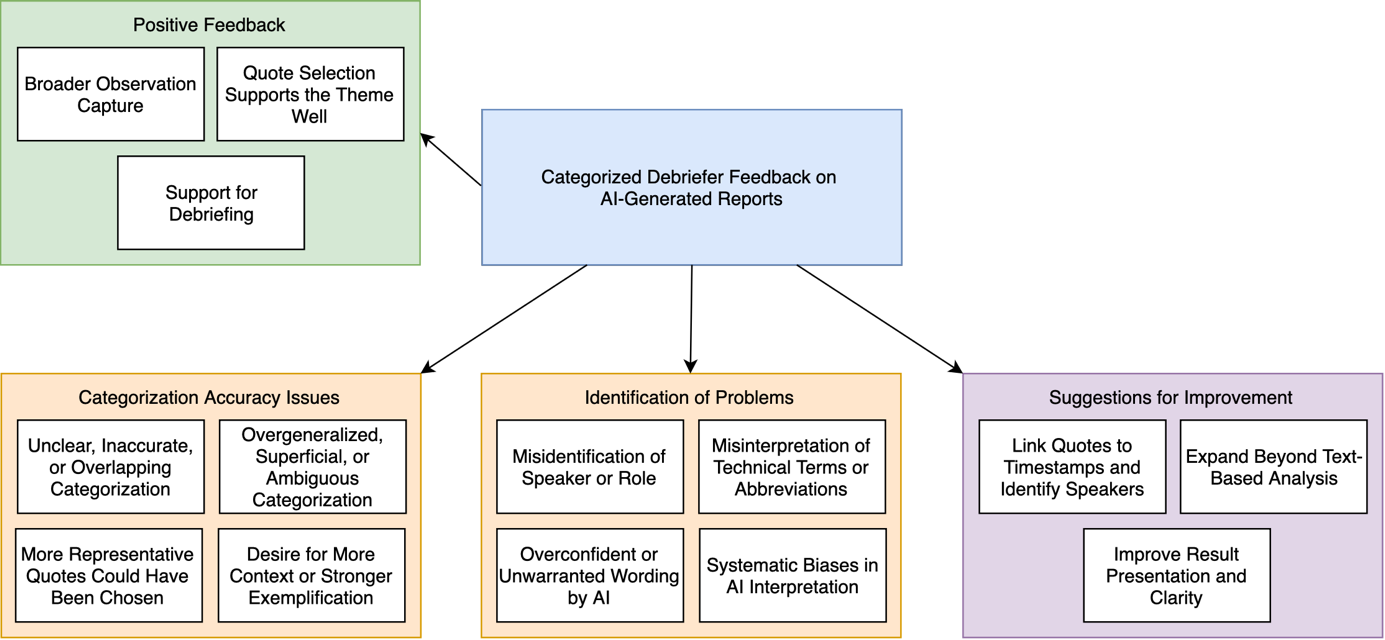


**Supplementary Figure S2:** Categorized debriefer feedback on AI-generated reports. Feedback clustered into four themes: positive feedback, categorization accuracy issues, identification of problems, and suggestions for improvement.

**
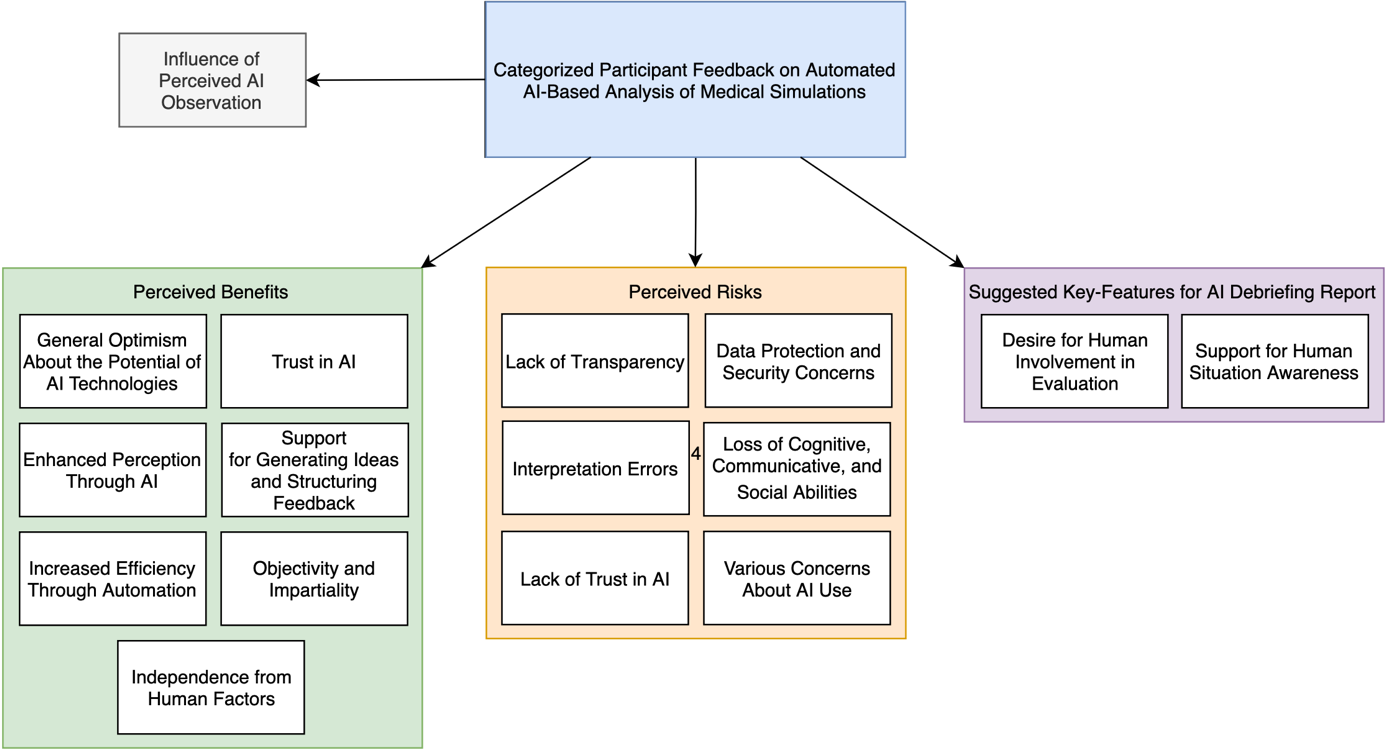
**

**Supplementary Figure S3:** Categorized participant feedback on automated AI-based analysis of medical simulations. Feedback was grouped into four main themes: perceived benefits, perceived risks, suggested key features for AI report and influence of perceived AI observation.

**Supplementary Table S2:** Categories and complete quotes from debriefers. The table presents key themes, subthemes, and illustrative comments from simulation debriefers on the quality and usefulness of AI-generated reports. Red highlights Chat-GPT and blue Sapient AI.

| **Theme** | **Subtheme** | **AI Text** | **Debriefer comments** |
| --- | --- | --- | --- |
| **Positive Feedback** | Broader Observation Capture | **Sapient AI: Sapient: Recognizing the Importance of Teamwork** The importance of teamwork was recognized, as illustrated by the quote: "SPEAKER_01: Then we’ve got it." This shows that the team members are working together to reach a solution. | I don’t remember the quote, but they called for help early and stood close together. |
|  |  | **Chat-GPT: Structured Communication**  Clear and structured communication is demonstrated multiple times:   - *"This is an emergency."* (06:36–06:37) – precise wording in critical situations. - *"Saturation becomes the focus."* (18:20–18:21) – updates are given using clear terminology. - *"Now we obviously have an airway."* (18:39–18:42) – a conclusion is drawn to establish the current status.   **Chat-GPT: Reflection and Debriefing** There are indications of reflection on the current experiences, as seen in: *"Then I would now say, if it’s an anticipated difficult airway, it might not really be a teaching situation either."* and *"But now we simply have to see if we can somehow cancel the operation, I think."* | I didn’t hear the first example, that would be a great example; always some short joint coordination, I didn’t hear concrete tools...  In practice it’s actually not possible to note so much, because you have to do several other things at the same time. That’s why this script would help me recognize priorities for the debriefing. |
|  |  | **Chat-GPT: Closed-Loop Communication** Messages were correctly received and acknowledged, as seen in: *"The scenario continues."*, *"Okay."*, and *"Intubation equipment is ready."* | Good examples, I didn’t even notice them. |
|  |  | **Chat-GPT: Structured Communication**  Clear and structured communication is demonstrated multiple times:   - *"This is an emergency."* (06:36–06:37) – precise phrasing in critical situations. - *"Saturation is becoming the focus."* (18:20–18:21) – updates are provided using clear terminology. - *"Now we obviously have an airway."* (18:39–18:42) – a conclusion is drawn to establish the current situation. | I didn’t hear the first example, that would be a great example; always some short joint coordination, I didn’t hear concrete tools... |
|  |  | **Chat-GPT: Closed-Loop Communication**  Responses to instructions and information are mostly complete:   1. *"Suction, please."* – *"Yes, of course."* (05:16–05:17). 2. *"Can you get the defib pads?"* – *"Yes."* (05:27–05:41). 3. *"Then another milligram of adrenaline."* – *"Okay, adrenaline coming."* (09:19–09:22). | Examples fit. (But I don’t remember them — which doesn’t have to mean anything — lots of noise in the control room; unrest in the scenario). That would be brilliant if CLC could be captured like that!!! |
|  |  | **Sapient AI: Reflection and Debriefing** There are indications of reflection: *"SPEAKER_01: That would have been mean."* and *"SPEAKER_01: We're not allowed to open the device because of the simulation."* These statements show that the team is reflecting on the experience and learning from the simulation. | Examples don’t fit from my point of view — I didn’t notice any examples for reflecting, but I generally had difficulties understanding it acoustically. |
|  |  | **Chat-GPT: Mutual Trust**  The interactions demonstrate trust in the abilities of team members:   1. *"Should I plug this in?"* – *"That's a socket."* (01:07–01:12). 2. *"Just come quickly, please, because it’s a patient."* (05:02–05:06). 3. *"I can’t get through."* – *"But I can still get through to suction tracheally, right?"* (08:18–08:27). | Not so bad at all... maybe AI can operationalize this better than humans... |
|  | Quote Selection Supports the Theme Well | **Chat-GPT: Structured Communication** The communication was structured, as clearly shown by the instructions: *"I’m switching to manual now."*, *"I’ll go get the airway cart."*, and *"And I should bring the fiber-optic cart too?"* | ***Correct*** |
|  |  | **Chat-GPT: Closed-Loop Communication** Messages were correctly received and acknowledged, as seen in the dialogues: *"Yes, that’s what she said."*, *"Yes, CO₂ is coming."*, and *"Good, we’re still on gas."* | ***Good examples*** |
|  |  | **Chat-GPT: Creating a Psychologically Safe Environment** There were several indications that team members felt free to speak up, such as: *"But be careful, please."*, *"I’m trying to ventilate."*, and *"Can you quickly hand over the Guedel tube?"* | *These quotes could be interpreted as indicating a certain psychological assurance."* |
|  |  | **Chat-GPT: Asking Clarifying Questions** Clarifying questions were asked to share knowledge and avoid misunderstandings, such as: *"Is Sufenta okay for you?"*, *"They’re looking for a senior consultant. They’re looking."*, and *"Is the tongue swollen?"* | ***correct*** |
|  |  | **Chat-GPT: Reflection and Debriefing** There were indications of reflection and debriefing to learn from the simulation, such as: *"We still need a reassessment."*, *"So, A is still a bit swollen."*, and *"In D we’re now determining the half-time."* | ***correct*** |
|  |  | **Chat-GPT: Asking Clarifying Questions** Clarifying questions were asked, such as: *"Are you pressing somewhere right now?"*, *"Let’s see, I’m not even sure—was he intubated using RSI?"*, and *"Do we have CO₂ now?"*, indicating an effort to avoid misunderstandings. | ***correct*** |
|  |  | **Chat-GPT: Sharing Unique Information** Crucial information was shared, such as: *"This is Mr. Rutschmann, he was intubated while jumping."*, *"He was wedged in with 4 watts of Propofol."*, and *"The positioning nurse had just—well, we were actually already ready, and during positioning, he pulled out the tube."* This shows that important information was communicated within the team. | ***correct*** |
|  |  | **Chat-GPT: Optimizing Shared Mental Models** There are indications of a shared understanding, such as: *"Now we probably need to deepen a bit."*, *"He has a known difficult airway, right?"*, and *"We don’t even know how much relaxant has actually taken effect."* This suggests coordination and alignment within the team. | ***correct*** |
|  |  | **Chat-GPT: Mutual Trust** Mutual trust was established, as seen in: *"name, next is LMA."*, *"Then let’s try the LMA."*, and *"name, could you keep an eye on the patient’s vital signs while we’re working here?"* This shows that tasks were delegated with confidence and trust. | ***correct*** |
|  |  | **Chat-GPT: Structured Communication** Structured communication is demonstrated through clear instructions such as *"You get the intubation cart"* and *"name, please call the OR nursing staff."* | ***Correctly identified, good match*** |
|  |  | **Chat-GPT: Sharing Unique Information** Crucial information was shared, such as *"She has such poor mucosa"* and *"65-year-old patient, preoperatively medicated with Dormicum, and now we have a saturation of 85."* | ***Correctly identified, supports structured communications*** |
|  |  | **Chat-GPT: Mutual Performance Monitoring**  Monitoring and feedback are present: *"name, let us know when we can administer the medication."* (10:01–10:06). *"Saturation is no longer measurable."* – *"Good, perfect."* (07:48–07:49). *"How do the pupils look?"* – *"Isotropic"* (13:23–13:30). | ***Helpful quote, correct*** |
|  |  | **Chat-GPT: Mutual Trust**  Trust is built through clear role assignments and feedback: *"name is at the head, with me as backup."* (06:38–06:41). *"name, we need a tube now."* – *"A tube, yes."* (08:56–08:59). *"name, you know this best."* (12:35–12:39) | ***Good examples*** |
|  |  | **Chat-GPT: Recognizing the Importance of Teamwork**  The central role of teamwork is emphasized multiple times, for example in task allocation: *"name, set up the access. name, are you ready?"* (05:30–05:36). It is also clear that the team works interdisciplinarily by involving specific professionals such as surgeons and OR nursing staff: *"name, please call the OR nursing staff."* (05:45–05:49). A clear understanding of the importance of interprofessional collaboration is also evident in the discussion of alternative options: *"Should we look for alternative specialties who could perform a surgical airway?"* (06:32–06:35). | ***These quotes fit better than other ones.*** |
|  |  | **Chat-GPT: Creating a Psychologically Safe Environment**  There are indications that team members can express their opinions:   - *"Now we need to make a plan first. So, what are your thoughts?"* (04:16–04:20) - *"What can we optimize about the airway?"* (05:49–05:51) - *"Should we switch Sugi?"* – *"Yes, gladly."* (11:46–11:48) The atmosphere appears supportive, as ideas are gathered and discussed constructively. | ***Correct, good quotes*** |
|  |  | **Chat-GPT: Structured Communication**  Communication is structured through checklists and clear protocols:   - *"We have a quick checklist, right?"* (07:06–07:09). - The plan is repeated several times: *"First plan: awake fiberoptic intubation. Second plan: intubation with D-blade. Third plan: surgical tracheotomy."* (07:09–07:30). | ***Good observation, the fact that the answer was included in the output was helpful. I could see if the participants noticed the question.*** |
|  |  | **Chat-GPT: Closed-Loop Communication**  Messages are confirmed and acted upon:   - *"name, we need a tube now."* – *"A tube, yes."* (08:56–08:59). - *"name, do you have catecholamine?"* – *"I have norepinephrine."* – *"Good, then we’ll give 5.5 adrenaline."* (11:08–11:14). - *"Did everyone hear that?"* – *"ENT is taking time, OR nursing staff is coming."* (06:26–06:32). | ***I highlighted these topics in the debriefing without knowing this transcript, very good.*** |
|  |  | **Chat-GPT: Asking Clarifying Questions**  Asking questions is a central element:   - *"What can we optimize about the airway?"* (05:49–05:51). - *"How do the pupils look?"* (13:17–13:24). - *"Do we actually have high-flow here?"* (01:52–01:55). | ***Very cool.*** |
|  |  | **Chat-GPT: Optimizing Shared Mental Models**  The team works to establish a common understanding:   - *"Let’s say it again for everyone together."* (07:04–07:06). - *"name, give me the fiberoptic and we need to set this up here."* (07:55–07:57). - The repeated planning and confirmation (*"Is everyone in agreement?"*) demonstrate the effort to align on a common approach. (09:18–09:20) | ***Good points*** |
|  |  | **Chat-GPT: Structured Communication**  Structured communication protocols are consistently applied:   1. *"We now have slightly increasing ventilation pressures"* (01:20) precisely describes the situation. 2. *"With an LMA, we are ventilated, we have CO₂, we have a slightly high ventilation rate"* (10:01) provides an overview of the patient’s status. 3. *"Then relax, absolutely please"* (04:00) is a clear, prioritized instruction. | ***I also noted these points*** |
|  |  | **Chat-GPT: Closed-Loop Communication**  Messages are correctly acknowledged and acted upon:   1. *"name, can you check if the positioning nurse really called name?"* – *"That’s one thing."* (03:30–03:39). 2. *"Draw up adrenaline once?"* – *"Here, just draw this up."* (06:30–06:44). 3. *"I need a little more."* – *"Yes, I think it’s not going in."* (04:17–04:21). 4. *"He was definitely awake because the infusion was para, right?"* (09:00). | ***These dialoges were observed by myself. It would be even nicer to have them linked to certain participants via AI.*** |
|  |  | **Chat-GPT: Asking Clarifying Questions**  Clarifying questions are frequently asked to avoid misunderstandings:   1. *"Let’s see, I don’t even know, was he RSI-intubated?"* (02:14). 2. *"Why do I have…? Ah, okay."* (03:26–03:30). 3. *"Should we quickly do ABCDE?"* (09:54). | ***Good*** |
|  |  | **Sapient AI: Mutual Performance Monitoring** Team members monitor each other and provide feedback, as seen in the discussion about the correct placement of equipment: *"SPEAKER_06: Make sure everything is already in place."* | ***If Speaker 6 was a participant, this is very good.*** |
|  |  | **Sapient AI: Optimizing Shared Mental Models** There are indications of a shared understanding within the team, as shown in the discussion about the need to intubate the patient: *"SPEAKER_07: Mask ventilation is not working."* | ***Good.*** |
|  |  | **Chat-GPT: Recognizing the Importance of Teamwork**  There are clear indications that team members understand the importance of teamwork. Roles and tasks were continuously assigned to manage the situation. Examples:   - *"Can you go to the head and try to ventilate with the mask?"* (01:41–01:51) shows dynamic task allocation. - *"name, we need help in OR8 with the patient."* (04:56–05:01) indicates involving external teams to resolve the situation. However, uncertainties like *"Should I set up the IT or what do we do now?"* (02:40–02:46) show that clear prioritization is sometimes lacking. | ***The first two examples i like very much.The second point about IT is not negative. Missing prioritization was also noted.*** |
|  |  | **Chat-GPT: Creating a Psychologically Safe Environment**  There are moments that show a climate was created enabling questions and support:   1. *"What should I prepare for you?"* (02:08–02:09) demonstrates openness to being involved in decision-making. 2. *"Should I slide a little more again?"* (03:53–03:55) suggests that proposals are made without hesitation. 3. *"Can you get the defibrillator pads?"* – *"Yes."* (05:27–05:41) shows acceptance and prompt execution of instructions. | ***Not bad.*** |
|  |  | **Chat-GPT: Asking Clarifying Questions**  The team asks targeted questions to resolve uncertainties:   1. *"What should I prepare for you?"* (02:08–02:09). 2. *"Should I trigger a rehab otherwise?"* (04:34–04:36). 3. *"Does she still have a pulse?"* (05:27–05:28). | ***I share the Ais interpretation.*** |
|  |  | **Chat-GPT: Optimizing Shared Mental Models**  The team works to develop a common understanding of the situation:   1. *"For everyone who just arrived, patient is in the airlock..."* (07:10–07:26). 2. *"Okay, how long have we been at it now?"* (08:33–08:59) shows efforts to synchronize the timeframe. 3. *"We now have a secured airway, she has a pulse again."* (09:37–09:44). | ***Good examples, I agree. We would need the fitting debriefing statements.*** |
|  |  | **Chat-GPT: Mutual Performance Monitoring**  There are clear signs that team members monitor and support each other:   1. *"Excuse me, watch your hands."* (03:55–03:56). 2. *"Do we have a pulse?"* (09:00–09:00). 3. *"But still, press again, we need to press more."* (09:24–09:27). | ***Good examples*** |
|  |  | **Chat-GPT: Reflection and Debriefing**  There are signs of reflection during the simulation, but no structured debriefing:   1. *"This might also not be the best teaching situation."* (03:27–03:35) shows self-reflection on the challenges. 2. *"But now we just have to see if we can somehow stop the operation, I think."* (09:44–09:49). 3. *"We are doing compressions, have 1 milligram of adrenaline in, and haven’t made much further progress yet."* (07:26–07:35) shows an assessment of the progress. | ***Very good examples*** |
|  |  | **Sapient AI: Recognition of the Importance of Teamwork** The importance of teamwork is clearly demonstrated through ongoing communication and coordination among team members. For example, the statement: *"Can you inform the doctors?"* – *"They look pretty blue."* (SPEAKER_05 / SPEAKER_00) illustrates mutual coordination and awareness of the necessity for joint action. | ***good*** |
|  |  | **Sapient AI: Creating a Psychologically Safe Environment** There are indications that team members were able to express themselves freely, as shown by statements like: *"You need to come now."* (SPEAKER_00) and *"I have the feeling that we need to sink somewhere in the center where we have no residue at all."* (SPEAKER_04) | ***This was also my impression. The team from the PACU didn't say much. Why? I think the attending was very team-oriented.*** |
|  |  | **Sapient AI: Closed-Loop Communication** There are examples of closed-loop communication: *"SPEAKER_00: They’re coming, right?"* *"SPEAKER_00: Yes."* and *"SPEAKER_04: Yes, for you."* | ***I also noted multiple examples of CLC*** |
|  |  | **Sapient AI: Sharing Unique Information** Critical information was shared: *"SPEAKER_00: She has a tongue… tongue carcinoma."* and *"SPEAKER_00: It was an 84% saturation."* 3. *"Then let’s see if there is any organ damage."* (12:42–12:46) emphasizes the focus on potential consequences. | ***Good examples*** |
|  |  | **Chat-GPT: Recognizing the Importance of Teamwork**  The communication clearly shows that the team members are aware of the importance of teamwork. The handover of information and mutual support are central:   - *"I’ll try it and you handle the suction."* (16:03–16:07) – demonstrates awareness of interdependent collaboration. - The dialogue between team members reflects a clear understanding of interprofessional roles, especially involving the senior physician (*"The senior physician wanted you to come now."* 06:33–06:36). | ***I also recognised this. Help was called right away, also surgery.*** |
|  |  | **Chat-GPT: Creating a Psychologically Safe Environment**  There are indications that team members feel free to express themselves:   - *"Can you connect me with the URL that’s with Mrs. Tobler?"* (05:53–06:00) – shows that questions can be asked. - *"Try to get a grip on her."* (07:22–07:27) – suggestions are openly shared. - *"Should I try to push a little?"* (14:12–14:22) – team members feel safe making proposals. | ***I concur with the AI. I heard multiple questions and suggestions. Thanks to the Attending for pushing Adrenaline.*** |
|  |  | **Chat-GPT: Closed-Loop Communication**  Messages are often confirmed and executed:   - *"Oxygen saturation is coming." – "Ah, now it’s coming."* (18:20–18:23). - *"10 micrograms of adrenaline?" – "It’s in."* (15:34–15:36). - *"Good, I have everything in." – "Gladly."* (09:38–09:40) | ***I love these examples.*** |
|  |  | **Chat-GPT: Asking Clarifying Questions**  Clarifying questions are present to avoid misunderstandings:   - *"How much propofol do you want?"* (09:14–09:15). - *"Can we ventilate her now?"* (10:22–10:30). - *"How is the ventilation now?"* (12:33–12:34). | ***Good.*** |
|  |  | **Chat-GPT: Sharing Unique Information**  Critical information is effectively shared:   - *“She is an ORL patient.”* (05:29–05:31) – communicates the patient background to the team. - *“You only see bloody, slightly bleeding tumor tissue.”* (11:28–11:30) – observations are directly shared. - *“Base of tongue carcinoma, planned.”* (02:52–02:56) – context for the plan is highlighted. | ***The second statement was from an instructor, not a participant. The AI misdetected this.*** |
|  |  | **Chat-GPT: Optimizing Shared Mental Models**  A common understanding is promoted:   - *“We have one minute now, we have given a situation update.”* (11:14–11:17) – clarifies priorities. - *“We can obviously ventilate now, so let’s do an A, B, C together.”* (18:39–18:42). - *“The patient is bradycardic and hypotensive.”* (15:12–15:29) – status updates create clarity. | ***I share the opinion. The attending contributed to the planning. What has to be done when?*** |
|  |  | **Chat-GPT: Reflection and Debriefing**  Reflection occurs in real time, but there is no evidence of structured debriefings:   - *“As it looked, after the 80% saturation, it rather dropped a bit.”* (06:50–06:55) – analysis of the situation. - *“I have the feeling we need to go somewhere where we have no residual at all.”* (03:28–03:32) – reflection on shortcomings. - *“Then we need to look again at how we conjuncture.”* (13:05–13:09) – preparation for the next step. | ***Perfect.*** |
|  |  | **Chat-GPT: Creating a Psychologically Safe Environment**  There were signs that team members freely expressed themselves and requested help:   1. "Can you quickly hand me the belt?" (02:41–02:44) shows trust in asking for support. 2. "May I get that?" (02:19–02:20) reflects an open atmosphere for coordination. 3. "Should we get more help?" (08:09–08:11) indicates willingness to involve external assistance. | ***Good examples*** |
|  |  | **Chat-GPT: Closed-Loop Communication**  The communication often included feedback, such as:   1. "Alright, we’re still on gas." – "Turn off the gas, right?" – "Then I’ll go to 4 here." (01:38–01:45) demonstrates successful feedback. 2. "Are you relaxed?" – "Yes." (03:03–03:07) 3. "Then I’ll prepare the fiber optic." – "Okay, good." (04:05–04:13) also shows confirmation. | ***Nice examples with the answers*** |
|  |  | **Chat-GPT: Sharing Unique Information**  Important information was shared:   1. "The positioning staff pulled out the tube." (02:30–02:34) identified a key cause. 2. "The infusion came out." (09:50–09:54) provided relevant details. 3. "Propofol is at 6." (09:27–09:28) supported medication monitoring. | ***Good examples*** |
|  |  | **Chat-GPT: Optimization of Shared Mental Models**  The team worked on creating a shared understanding:   1. "So A, still a little swollen." (11:45–11:49) systematically checked the situation. 2. "We still need a reassessment." (11:38–11:44**)** emphasized the priority of joint evaluations. | ***Super*** |
|  |  | **Chat-GPT: Mutual Trust**  Trust among team members was evident:   1. "Can I help you?" – "Yes." (06:22–06:23) shows spontaneous support. 2. "Can you lower the table a bit more?" (07:42–07:45) demonstrates assistance during operative tasks. 3. "Are you in?" – "No, missed again." (06:23–06:24) shows openness in acknowledging mistakes. | ***1 = good***  ***2 = not fitting***  ***3 = good*** |
|  |  | **Sapient AI: Optimization of Shared Mental Models** SPEAKER_03 provides an overview of the patient’s condition: "We are intubated. For the induction, however, he was awake under the fiberoptic." SPEAKER_02 summarizes the situation: "We have an airway problem." SPEAKER_02 gives a clear instruction: "Then you do Adam’s apple palpation, cut once two centimeters down." | ***Very beautiful examples*** |
|  |  | **Sapient AI: Asking Clarifying Questions** SPEAKER_01 asks about the necessity of an action: "And why head down?" SPEAKER_00 inquires about the desired position: "How much should we raise the head?" SPEAKER_02 asks about the status: "Can you ventilate?" | ***I used these points during the debriefing. Very nice observations.*** |
|  |  | **Sapient AI: Recognizing the Importance of Teamwork** The importance of teamwork becomes clear in the transcript when SPEAKER_03 addresses task distribution: "Then you lead anesthesia and we assist, right?" and SPEAKER_04 responds: "As usual, of course." This shows that team members understand their roles and collaborate effectively. | *Regarding the point 'recognizing the importance of teamwork,' I find Speaker 03's statement very insightful and valuable. We also considered this aspect to be particularly important for the teamwork observed and addressed it accordingly during the debriefing.* |
|  |  | **Sapient AI: Creating a Psychologically Safe Environment** SPEAKER_00 feels safe enough to admit being new on the job: "I’m new at this." SPEAKER_01 asks for help: "Can you please call the attending?" and SPEAKER_00 admits a mistake: "I’m a clumsy man." | *These points are nicely emphasized, even if in this case they were mentioned by the ESP. What is missing here is a representation of the tone of voice, which would reflect the calm and controlled aspect of psychological safety. This is a dimension that occurs largely 'between the lines' and non-verbally, and it is therefore not surprising that it is not prominently represented. As instructors, we pay particular attention to this aspect.* |
|  |  | **Chat-GPT: Recognizing the Importance of Teamwork** The importance of teamwork was acknowledged, as shown by the statement, "*name*, can you check if the positioning nurse really called *name*?" This demonstrates that tasks were delegated and team members involved in the process. | ***Good examples*** |
|  |  | **Chat-GPT: Asking Clarifying Questions** Clarifying questions were asked, such as "Are you pressing anywhere right now?" and "Let's see, I don’t even know, was he RSI-intubated?" as well as "Do we have CO2 now?", indicating an effort to avoid misunderstandings. | ***OK*** |
|  |  | **Chat-GPT: Optimizing Shared Mental Models** There are indications of a shared understanding, such as "Now I think we need to deepen a bit," and "He has a known difficult airway, right?" as well as "We don’t even know how much relaxant has actually taken effect," all pointing to team alignment. | *The information can indeed help establish and continuously update a shared mental model within the team. However, I find the planning aspect somewhat lacking—something along the lines of: what comes next, what follows after that… and finally checking if there are any questions or comments, etc.* |
|  |  | **Chat-GPT: Mutual Trust** Mutual trust was established, as shown by statements like "*name*, next is the LMA," and "Then try the LMA," as well as "*name*, can you keep an eye on the patient’s vital signs while we’re fiddling around here?" This demonstrates that tasks were delegated with confidence.. | ***Fair enough*** |
|  |  | **Chat-GPT: Mutual Performance Monitoring**  Examples of mutual monitoring: "Make sure everything is already available." (00:31–00:33), "Armboards on, everything attached?" (00:22–00:23), "The saturation is rising." (07:18–07:24) | ***1 and 2 ok, 3 is not that well fitting*** |
|  |  | **Chat-GPT: Optimization of Shared Mental Models**  There were indications of shared mental models: "Make sure everything is already available." (00:31–00:33) ➔ Team preparation, "Tongue. No epiglottis, no vocal cords." (04:23–04:24) ➔ Clear status description.  "CO2, CO2, CO2. The saturation is rising." (07:18–07:24) ➔ Shared understanding of the patient’s condition. | ***Very nice examples*** |
|  |  | **Chat-GPT: Recognizing the Importance of Teamwork**  The team demonstrates a strong awareness of the importance of collaboration, especially in emergency situations:   - *“Who’s at the head?” – “I’m at the head.”* → The clear role assignment shows that team members know and carry out their tasks. - *“Are you watching the circulation and anesthesia?”* → This statement highlights the importance of interprofessional collaboration, with anesthesia and nursing working closely together. | ***Nice examples and well fitting*** |
|  |  | **Chat-GPT: Creating a Psychologically Safe Environment**  Examples show that team members can admit mistakes or ask questions:   - *“Oh no, now I’ve... oh, oh, oh.”* → A team member openly admits a mishap. - *“Is the patient relaxed?”* → Indicates that questions can be asked without fear of criticism. - *“Maybe the Voice of God can give us some input here.”* → A call for clarification shows trust in external support.. | ***Good examples*** |
|  |  | **Chat-GPT: Structured Communication**  Clear and structured communication was frequently evident:   - *“We have an A problem and a C problem. Please come.”* → Clear identification of issues for precise feedback. - *“Adam’s apple, cut two centimeters down.”* → Precise instructions during the cricothyrotomy. - *“A. We performed a coniotomy. We have CO2.”* → Structured debriefing after securing the airway. | *The mention that structured communication was frequently observable in this scenario is particularly important and accurate. This has been highlighted wonderfully. The examples used to support this point are also ideal and reinforce the statement effectively. This aligns very well with the key elements of our debriefing.* |
|  |  | **Chat-GPT: Closed-Loop Communication**  Messages were accurately received and confirmed:   - *“Please call the senior physician.” – “I’m calling the senior doctor.”* → Clear command executed and acknowledged. - *“Can we ventilate?” – “No, it’s not possible.”* → Precise feedback to critical questions. - *“Do we have CO2?” – “CO2 is coming.”* → Confirmation of a successful intervention. | ***Very nice examples*** |
|  |  | **Chat-GPT: Asking Clarifying Questions**  Clarifying questions were used to avoid misunderstandings:   - *“How much should we raise the head?”* → Specification when instructions were unclear. - *“Using the D-Blade, does that work for you?”* → Clarification of equipment choice. - *“How deep do you want to go in there?” – “6 cm.”* → Preventing errors through clear communication. | ***Very good examples*** |
|  |  | **Chat-GPT: Sharing Unique Information**  Crucial information was shared contextually:   - *“The positioning assistant pulled out a Venflon and a tube.”* → Critical information for planning the next steps. - *“We have bradycardia.”* → Early warning about the circulatory status. - *“The intubation was fiberoptically difficult during bariatric surgery.”* → Background information to guide appropriate actions. | ***good*** |
|  |  | **Chat-GPT: Mutual Trust**  Trust within the team was evident:   - *“Please prepare everything for the cricothyrotomy for me.” – “Yes.”* → Confidence in carrying out the instruction. - *“I’m going to intubate. Will it work?” – “You can do it.”* → Encouragement in a critical situation. - *“Can you ventilate?” – “Wait, I’m trying.”* → Open communication without fear of failure. | ***The second is the best example*** |
|  |  | **Chat-GPT: Reflection and Debriefing**  Reflection was evident:   - *“We’re reviewing everything: A. We performed a cricothyrotomy.”* → Systematic check following the procedure. - *“We now somehow have to move back from the cricothyrotomy to the tube.”* → Forward-looking planning of the next steps. - *“We’re going to do a bronchoscopy now to take a look up there.”* → Reflection and strategy adjustment based on previous observations. | ***Very good examples, especially for gathering the participants initial thoughts during the debriefing.*** |
|  |  | **Chat-GPT: Recognizing the Importance of Teamwork**  The team clearly acknowledges the interprofessional roles and their significance:   - *“name, can you check if the positioning nurse really called name?”* (03:30) shows trust in task delegation. - *“We need, name, can we get someone else to help…?”* (05:22) demonstrates willingness to involve external support. - Despite the stressful situation, the team strives to act in a coordinated way, for example through clearly defined responsibilities like securing the airway and administering medication. | ***Good examples*** |
|  |  | **Chat-GPT: Creating a Psychologically Safe Environment**  There are indications that team members communicate openly and feel safe:   1. *“Are you pressing somewhere right now, or not?”* – *“No?”* (01:10–01:14) shows that clarifying questions are asked without fear of consequences. 2. *“I don’t know why I…”* – *“Am I already in?”* (12:52–13:04) demonstrates that uncertainties can be expressed. 3. *“What’s alarming now again?”* – *“Do we need to pay attention to this alarm?”* (11:59–12:08) indicates that questions are accepted and answered. | ***I share these thoughts*** |
|  |  | **Chat-GPT: Sharing Unique Information**  Crucial information is shared to create a common understanding:   1. *“The tube is secured again, but everything feels so flexible.”* (00:22) 2. *“Now I think we need to deepen a bit.”* (02:58) | ***Ok good*** |
|  |  | **Chat-GPT: Optimizing Shared Mental Models**  The team develops a common understanding of the situation:   1. *“With a LMA, we are ventilating, we have CO2, and currently a slightly high ventilation rate.”* (10:01) | *I agree; this would also include 'talking to the room,' verbalizing, sharing information without being explicitly asked.* |
|  |  | **Chat-GPT: Reflection and Debriefing**  There are indications of reflective considerations:   1. *“Maybe it really was some kind of reaction like bronchospasm or something else.”* (10:35) 2. *“But to what? There actually was no trigger, I don’t know.”* (10:43) 3. *“We can think about that again afterwards.”* (10:49) | ***Interesting points, did not notice that.*** |
|  |  | **Sapient AI: Recognizing the Importance of Teamwork** The importance of teamwork was recognized, as reflected in the quote: "SPEAKER_01: Then we have it so far." This demonstrates that team members collaborate effectively to reach a solution. | ***Good speak-ups.*** |
|  |  | **Sapient AI: Creating a Psychologically Safe Environment** There are indications that team members feel free to express themselves, as seen in the following quotes: "SPEAKER_01: That hurts. That’s actually good." and "SPEAKER_00: That wasn’t so good." These statements show that team members openly share their assessments. | ***Good.*** |
|  |  | **Sapient AI: Closed-Loop Communication** There are examples of closed-loop communication: "SPEAKER_01: I'm starting the video. SPEAKER_01: You need to press OK here. SPEAKER_01: I did." This shows that messages are received, confirmed, and executed. | ***Can be, I did not hear that much CLC*** |
|  |  | **Sapient AI: Asking Clarifying Questions** Clarifying questions are asked, as seen in the quotes: "SPEAKER_01: Do you want to draw up anything else? Other medications?" and "SPEAKER_01: Do you want a stylet?" These questions help share knowledge and prevent misunderstandings. | ***Good*** |
|  |  | **Sapient AI: Sharing Unique Information** Critical information is shared, as seen in the quotes: "SPEAKER_01: She said she registered it incorrectly." and "SPEAKER_01: The tube was pulled out during positioning." These pieces of information are important for understanding the situation. | ***Many updates were exchanged. Good.*** |
|  |  | **Sapient AI: Optimizing Shared Mental Models** There are indications of a shared understanding within the team: "SPEAKER_01: We are now reorienting the video." and "SPEAKER_00: We're in, we can see it." These statements demonstrate that the team is aligned and working with the same situational awareness. | ***Good examples*** |
|  |  | **Sapient AI: Mutual Performance Monitoring** Team members monitor each other: "SPEAKER_00: Saturation 84, right?" and "SPEAKER_01: CO2 is 7.78." This monitoring helps ensure high-quality care.  **Closed-Loop Communication**  There are several examples of successful closed-loop communication:   - *"Airway."* — *"We actually haven’t checked that yet. Good input."* — The input is immediately acknowledged and acted upon. - *"Stethoscope, please."* — *"We’re at the shore."* — Instructions receive prompt feedback. - *"Ventilate."* — *"Rising and falling."* — The success of the action is directly confirmed. | ***OK, I did not hear that much CLC*** |
|  |  | **Chat-GPT: Asking Clarifying Questions**  Clarifying questions are used to avoid misunderstandings:   - *"Did she respond to you before?"* — This question seeks to clarify the patient’s condition. - *"May I ask what you're doing right now?"* — Questions about ongoing measures promote shared understanding. - *"What’s coming over the floor of the mouth?"* — Clarifying questions help adapt the treatment strategy. | ***Regular questions, I heard a lot of them*** |
|  |  | **Chat-GPT: Sharing Unique Information**  Unique information is provided that the team may not yet have:   - *"Base of tongue carcinoma — it will probably look catastrophic, but we actually haven’t looked yet."* — Critical background information on the patient’s history. - *"She was in the recovery room, no longer responsive there. Low saturation."* — These details offer insight into the current clinical situation. - *"An iGel must be done with both hands."* — Important procedural information for task execution. | ***Good*** |
|  |  | **Chat-GPT: Optimizing Shared Mental Models**  The communication reflects the development of a shared understanding within the team:   - *"So, A is now intubated."* — Ensures that all team members are on the same page. - *"One push, and then we immediately start induction — that's my suggestion."* — A clearly communicated plan promotes alignment. - *"Propofol is running."* — Real-time updates help maintain a common mental model. | ***Examples are fitting*** |
|  |  | **Chat-GPT: Mutual Trust**  There are clear signs of mutual trust within the team:   - *"Do you want to do it?"* — Delegating tasks demonstrates confidence in colleagues’ abilities. - *"Shall we already... Now my question would be, since you're already handling the OLA..."* — Openly sharing concerns and suggestions shows psychological safety. - *"I'll take over here."* — Responsibility is assumed without hesitation, indicating trust in one another. | ***Attending lets the resident perform procedure, coaches, and supervises her. Very good.*** |
|  |  | **Chat-GPT: Mutual Performance Monitoring**  Team members monitor and support each other:   - *"Maybe quickly check the airway."* — Team members watch for potential gaps in the workflow. - *"Can you please prepare the strap?"* — Assistance with equipment preparation. - *"Once that’s done, we’ll start the induction immediately, that’s my suggestion."* — The team continuously reviews and confirms the next steps. | ***I find the examples fitting*** |
|  |  | **Chat-GPT: Recognizing the Importance of Teamwork**  The transcript demonstrates that team members understand the crucial role of collaboration, especially when managing complex situations. For example, during the difficult airway management, the exchange *"That’s a good input. Maybe quickly check the airway."* illustrates how team members value each other’s contributions. The responses to suggestions show that every member’s input is appreciated and that everyone actively contributes to patient care. | ***Good updates.*** |
|  |  | **Chat-GPT: Creating a Psychologically Safe Environment**  The transcript contains several indications that team members feel they are in a trusting and safe atmosphere where they can communicate openly:   - *"Good input. Maybe quickly enter the airway."* – A suggestion is acknowledged, signaling appreciation. - *"Shall we first try to take a look?"* – Questions and proposals are brought up without hesitation. - *"May I watch what you are doing now?"* – Open questions about ongoing actions show that uncertainties can be addressed without fear.   These examples demonstrate a culture where mistakes or uncertainties are openly discussed and addressed together. | ***Good examples for debriefing*** |
|  |  | **Chat-GPT: Optimization of Shared Mental Models** There are indications of a shared understanding within the team, as shown by statements like "We have no airway." and "She has stopped, we are performing compressions, 1 milligram of adrenaline administered." | ***Very good*** |
|  |  | **Chat-GPT: Asking Clarifying Questions** Clarifying questions were used, such as "Should I set up the IT or what are we doing now?" and "Do we have defibrillator pads?" | ***Good examples*** |
|  |  | **Chat-GPT: Creating a Psychologically Safe Environment** There are indications that team members feel free to speak up, as shown by statements like "Should I plug this in?" or "Do you need a peripheral IV?" Questions are asked and help is requested, suggesting a psychologically safe environment. | ***I noticed similar examples.*** |
|  |  | **Chat-GPT: Mutual Trust**  The trust among team members is clearly evident:   1. "I’m managing the line, the intubation is ready." This statement reflects confidence in a colleague’s abilities. 2. "I’m advancing the tube." – "Your hand is in the way." Open communication occurs here without blame. 3. "I’m holding the tube." This shows a strong sense of responsibility. | ***Good examples*** |
|  |  | **Sapient AI: Sharing Unique Information** Critical information is shared, such as:   - *"SPEAKER_01: ... who was planned for a base of tongue surgery."* - *"SPEAKER_03: This is probably a difficult airway."* | ***Good examples*** |
|  |  | **Sapient AI: Optimizing Shared Mental Models** There are indications of a common understanding within the team, such as:   - *"SPEAKER_02: Yes, so base of tongue carcinoma, it will probably look catastrophic, but we actually haven’t looked inside yet."* - *"SPEAKER_03: We are at... That fits roughly."* | ***ok*** |
|  |  | **Chat-GPT: Reflection and Debriefing**  The transcript contains only few indications of reflection. However, at the end, there is a brief moment of reflection:   1. "Even though I was holding the tube and then we had to pull it out of the hand." This shows awareness of potential areas for improvement. 2. "That would have been harsh." This could serve as a starting point for debriefing. 3. "We must not open this part because of the simulation." This statement indicates conscious reflection on the given conditions. | ***ok*** |
|  |  | **Chat-GPT: Asking Clarifying Questions**  Clarifying questions are actively used to avoid misunderstandings:   1. "Do you want to draw up anything else? Other medications?" This shows willingness to clarify details. 2. "Holding number 84, right?" This question verifies important data. 3. "Did you want the LMA or the video laryngoscope?" This ensures the correct equipment is being used.   **Chat-GPT: Creating a Psychologically Safe Environment**  There are several examples indicating that team members feel free to speak openly. For instance:   - *"We actually haven’t checked that yet."* - *"Good input."* - *"So, the Guedel airway, please."* | ***Good examples*** |
|  |  | **Chat-GPT: Closed-Loop Communication**  The team uses effective feedback to ensure that information is understood and executed. Examples:   1. "You’re starting the video, right?" – "I’m starting the video." Confirmation of the task. 2. "The line is in." – "Is it in?" – "Yes." This feedback ensures clarity. 3. "Do you have a Guedel airway?" – "No, we’ve inserted the IV." Here, clarity is provided through the response. | ***Very fitting*** |
|  |  | **Chat-GPT: Recognizing the Importance of Teamwork**  The transcript demonstrates functional teamwork, with interactions indicating clear task delegation and collaboration. The participants acknowledge that different roles are important to ensure smooth workflow. An example is the statement: “I’m calling *name* and getting... Bring me the fiberoptic scope that’s out in the hallway.” This shows that tasks were efficiently delegated to achieve the goal. | ***ok*** |
|  |  | **Chat-GPT: Creating a Psychologically Safe Environment**  There are indications that the team communicates openly despite high stress. For example:   1. “Do you want to draw up something else? Other medications?” This question shows that opinions and suggestions are welcomed. 2. “Okay, then I’ll get that.” This demonstrates a willingness to provide support. 3. “You can turn him once more so he goes the other way.” This indicates that team members help each other without fear of making mistakes. | ***Good debriefing material.*** |
|  | Support for Debriefing | **General comments** | ***Overall, I found the transcript with the timestamps very helpful yesterday. Today, both were not so helpful for me. But that could also be because of me. After the first impression yesterday, I think the concept has a lot of potential.***  ***What was quoted here, I find useful in many points. I would usually categorize it under other topics. But I think that does not really matter.***  ***I would benefit from such a transcript in a debriefing.***  ***Especially the note that structured communication was frequently recognizable in this scenario is important and correct. That is wonderfully highlighted. The examples used to support this statement are also ideal. This corresponds very well with the central elements of our conducted debriefing.***  ***I find this report significantly more helpful and concrete compared to the other; especially the timestamps are very helpful for the debriefing; some example sentences are partly too short for me or there is too little context information to make a valid statement (e.g. “okay, good”); another positive point about this report: there are more examples, in the other report it is always the same examples.***  ***Subjectively, I feel that overall the points raised, both good and missing, are much better covered and more precisely embedded in the context in this transcription than in the other version. It becomes clearer to me which situations are meant with the quotes. I could therefore imagine such support as a real added value for the debriefing.*** |
| **Categorization Accuracy Issues** | Unclear, Inaccurate, or Overlapping Categorization | **Chat-GPT: Creating a Psychologically Safe Environment**  Quote: "Oh, come on. Sorry." (06:13–06:14) ➔ This shows a willingness to acknowledge one's own mistakes. | *I find it contextually inappropriate here; I interpreted this "Come on" as an expression of stress/pressure, in the sense of "Come on, just do it, it has to work now," and not as a "sorry, I can't do it."* |
|  |  | **Chat-GPT: Sharing Unique Information** Quote**:** "Tongue. No epiglottis, no vocal cords." (04:23–04:24) ➔ Clearly formulated observations. | ***This may have been the VOG*** |
|  |  | **Chat-GPT: Mutual Trust**  Trust was evident in: **Quote:** "Should I still put it on?" Response: "Yes, that's right." (00:14–00:17) | ***This was not part of the scenario*** |
|  |  | **Chat-GPT: Mutual Performance Monitoring**  Team members monitor each other and provide feedback:   1. "Alex, could you please quickly place the IV line now?" (05:08). 2. "You could also do that otherwise." (09:48). 3. "Then I would start preparing everything now for the re-intubation." (09:18). | *For mutual performance monitoring, the other side is missing for me: the above examples are purely instructions, I am missing the reciprocity.* |
|  |  | **Chat-GPT: Closed Communication Loop**  Messages are correctly acknowledged and executed:   1. "*name*, can you check if the logistics nurse actually called *name*?" - "That's one thing" (03:30–03:39). 2. "Draw up adrenaline once?" - "Here, just draw it up" (06:30–06:44). 3. "I need a bit more." - "Yeah, I don't think it's going in" (04:17–04:21). | *In my opinion, none of the above statements are examples of closed-loop communication; confirmations are missing, the above examples are one-way addressed.* |
|  |  | **Sapient AI: Mutual Trust** Mutual trust is established, as seen in the quotes: "SPEAKER_01: I would just go ahead and adjust it now." and "SPEAKER_00: I’m holding the tube." These demonstrate that team members rely on one another. | *Hard to say, they spoke very closely together, all stood close to the head, and apparently helped each other.* |
|  |  | **Chat-GPT: Mutual Performance Monitoring**  Examples of mutual monitoring:   1. **Quote:** "Make sure everything is already in place." (00:31–00:33) 2. **Quote:** "Armboards on, everything on?" (00:22–00:23) 3. **Quote:** "The saturation is rising." (07:18–07:24) | ***Quote three is not relevant, one and two are.*** |
|  |  | **Sapient AI: Closed-Loop Communication** There are examples of closed-loop communication, such as: **SPEAKER_02:** "Inhalation with adrenaline." **SPEAKER_01:** "But did she respond to you before at all?" **SPEAKER_02:** "Only with groaning." | ***I did not here these.*** |
|  |  | **Sapient AI: Mutual Performance Monitoring** Team members monitor each other, as in: **SPEAKER_03:** "Doesn't look like anything is going in." and **SPEAKER_03:** "The thorax is holding us back." | *I don’t find these examples appropriate... I had the impression that the senior physician observed a lot and watched what the others were doing.* |
|  |  | **Sapient AI: Reflection and Debriefing** There are reflections on the experience, such as: **SPEAKER_04:** "Thank you very much for the scenario." and **SPEAKER_02:** "Let’s leave it like this for now, before I end up breaking the mannequin, right?" | *The examples do not fit because they came from the instructors – I did not perceive any reflection or subsequent joint evaluations..* |
|  |  | **Chat-GPT: Mutual Performance Monitoring**  The team monitors and supports each other:   1. **"We’re trying to ventilate."** This shows coordination to ensure success. 2. **"You can turn him again so that he goes in the other direction."** This helps to find an optimal solution. 3. **"So you can relax again in a moment,** *name***, so that we can... good."** This indicates that the team is monitoring performance and making necessary adjustments. | *In my view, the examples do not fit here. I couldn’t really see it well either because they all stood so close around the head. Hard to assess.* |
|  |  | **Sapient AI: Reflection and Debriefing** There are indications of reflection, such as: **SPEAKER_01:** "That would have been mean." and **SPEAKER_01:** "We're not allowed to open the device because of the simulation." These statements show that the team reflects on the experience and learns from the simulation. | *Examples don’t fit from my point of view—I didn’t notice any examples of reflection, but generally had difficulty hearing it clearly.* |
|  |  | **Chat-GPT: Mutual Trust**  Trust between team members was evident:   1. **"Can I help you?" – "Yes." (06:22–06:23)** shows spontaneous support. 2. **"Can you lower the table a bit more?" (07:42–07:45)** highlights assistance during procedural tasks. 3. **"Are you in?" – "No, missed again." (06:23–06:24)** demonstrates openness in acknowledging mistakes. | ***1 = good, 2 = not fitting, 3= good*** |
|  |  | **Chat-GPT: Reflection and Debriefing**  There are few explicit signs of structured reflection, but the following examples show engagement with the situation:   1. **"Did I do something wrong now? What should I do?"** (00:56–00:59) → Self-reflection and seeking guidance for improvement. 2. **"It won't get better just by saying the word."** (09:12–09:13) → Honest and realistic assessment of the situation. | ***These are not reflections but just observations.*** |
|  |  | **Chat-GPT: Structured Communication**  Communication is at times clearly structured, but often lacks precision:   1. **"For everyone who just joined, the patient is in the induction area…"** (07:10–07:26) summarizes the situation to bring new team members up to speed. 2. **"We’ve given 1 milligram of adrenaline."** (07:26–07:35) is a clear update on medication administration. 3. **"Look, those are the things for the infusion leak."** (02:33–02:36) reflects targeted instructions, though the wording is imprecise. | *The second example fits somewhat – communication is clear (dosage), but still not structured communication; the first example fits as well.* |
|  |  | **Chat-GPT: Creating a Psychologically Safe Environment** There are signs of a psychologically safe environment, such as: "Can you answer the phone, yes, exactly," and "Then we need a mask now," as well as "*name*, I’ll just leave him to that for now." These examples suggest that team members feel comfortable speaking up and asking for help. | *The psychologically safe environment was present. However, I did not see it reflected in these quotes.* |
|  |  | **Chat-GPT: Recognizing the Importance of Teamwork** The importance of teamwork was highlighted through coordinated collaboration and clear instructions, such as "Can you do that?" and "Can you please get a senior doctor?" The different roles within the team were made evident by task delegation and mutual support. | *The different roles within the team became apparent through task allocation and mutual support: However, in this scenario, the task allocation was not very clear to me. It would be interesting to understand on what basis the AI recognizes this.* |
|  |  | **Chat-GPT: Recognizing the Importance of Teamwork** The importance of teamwork was clearly acknowledged, as reflected in the statement "*name* from anesthesia and the anesthesia team," which emphasizes the collective effort and role distribution within the team. | ***Does not match*** |
|  |  | **Chat-GPT: Recognizing the Importance of Teamwork**  The team demonstrates clear recognition of interprofessional roles and their significance: "*name*, can you check if the positioning nurse actually called *name*?" (03:30) shows trust in task delegation. | *It rather shows that there is some mistrust towards the positioning nurse.* |
|  |  | **Sapient AI: Structured Communication** Structured communication is used, as shown in the quotes: **SPEAKER_01:** "Hi *name*, this is *name*, Room 6 in F-OR." and **SPEAKER_00:** "We can’t deepen the anesthesia, we can’t relax again." | *I also heard “So the next steps are...,” but it was interrupted... I didn’t hear much structured communication, nor any other examples of it.* |
|  |  | **Sapient AI: Mutual Trust** Mutual trust is fostered, as illustrated by statements like:   - "SPEAKER_02: That’s a good input." - "SPEAKER_03: Then first the finished edge." | *I can’t really judge, except perhaps for one example: the senior physician doesn’t perform the cricothyrotomy themselves but teaches a colleague and lets her do it.* |
|  |  | **Sapient AI: Mutual Trust** Mutual trust is built, as shown in: **SPEAKER_02:** "That’s a good input." and **SPEAKER_03:** "Then first the finished edge." | *Very similar examples to those under Closed-Loop Communication. Maybe CLC should really be defined through the loops themselves. Otherwise, the examples here are good.* |
|  |  | **Chat-GPT: Sharing Unique Information**  Critical information is shared to manage the situation:   1. **"The intubation is ready."** This information is essential to initiate the next step. 2. **"CO2 is coming."** This provides clear feedback on the success of ventilation. 3. **"Saturation number 84."** This information about oxygen saturation aids decision-making. | ***Does not match category*** |
|  |  | **Chat-GPT: Recognizing the Importance of Teamwork**  The importance of teamwork was recognized, as reflected in the statement "*name*, can you check if the positioning nurse actually called *name*?" This shows that tasks are delegated and team members are involved in the process. | ***Does not match category*** |
|  |  | **Chat-GPT: Reflection and Debriefing** Reflection and debriefing took place, as seen in statements like: "Now we can briefly say, we have anesthesia running, we have relaxed the patient, we have given adrenaline once, and now we have a ventilated patient with a LMA," and "But should we leave the situation like this for now, or should we try to get a tube in?" as well as "The only thing I don’t know is whether the patient was awake at any point or anything like that," which indicate an evaluation of the current situation and planning of next steps. | *For me represents structured communication and updating of the shared mental model; reflection and debriefing tend to take place after the case.* |
|  |  | **Chat-GPT: Creating a Psychologically Safe Environment** There are signs of a psychologically safe environment, such as: "Can you join in, *name*?" and "*name*, are you ready?" These show that team members feel comfortable contributing and confirming their willingness to participate. | *I would have rather categorized that under asking clarifying questions.* |
|  |  | **Chat-GPT: Closed-Loop Communication** There were closed communication loops, such as: "Then put to sleep, had 0.2 Fentanyl and 100 Esmeron," and "Then we need a mask now," as well as "And please, maybe you could call our senior physician," which show that messages were received and acknowledged. | *Yes, these sentences were said. I would have categorized them more as sharing information. It would be helpful here if the quotes were attributed to specific speakers.* |
|  |  | **Chat-GPT: Structured Communication**  Communication follows clear patterns and structures. Examples:   1. **"I’m handling the line, the intubation is ready."** This clearly signals who is responsible for which tasks. 2. **"CO2 is coming."** A precise and brief status update. 3. **"The tube is lubricated, the slit belt is ready."** The structured description of next steps facilitates collaboration. | *For me, these examples would point more toward Closed-Loop Communication. In this category, I would rather expect things like Time Out, 10 for 10, iSABR, etc., which I wasn’t able to observe.* |
|  |  | **Chat-GPT: Mutual Performance Monitoring**  The team monitored and supported each other:   - "I’m switching Propofol to gas and will do a bit of follow-up ventilation." → Collegial support during ventilation. - "Can you handle the rest, please?" → Tasks were delegated to increase efficiency. - "Are you watching circulation and anesthesia?" → Double monitoring of critical parameters. | *I find that a plausible line of reasoning, even though there’s significant overlap with communicative aspects.* |
|  |  | **Chat-GPT: Mutual Performance Monitoring** There was mutual performance monitoring, as seen in statements like: "*name*, can you check if the positioning nurse actually called *name*?" and "*name*, can you keep an eye on the patient’s vital signs a bit while we’re fiddling here?" as well as "Shall we quickly do an ABCDE assessment now?" These indicate ongoing monitoring and support within the team. | *“I’d quickly categorize ‘Shall we quickly do ABCDE then?’ under the importance of teamwork.”* |
|  |  | **Chat-GPT: Reflection and Debriefing** Reflection and debriefing took place, as illustrated by statements such as: "Now we can briefly say, we have anesthesia running, we have relaxed the patient, we have given adrenaline once, and now we have a ventilated patient with a LMA," and "But should we leave the situation like this for now, or should we try to get a tube in?" as well as "The only thing I don’t know is whether the patient was awake at any point or anything like that," which indicate evaluation of the current situation and planning of next steps. | *I would categorize "But should we leave the situation like this for now, or should we try to get a tube in?" under trust or valuing the opinions of others — as it invites input and shows openness to team perspectives.* |
|  |  | **Chat-GPT: Mutual Performance Monitoring** There was mutual performance monitoring, as shown in statements like: "*name*, can you check if the positioning nurse actually called *name*?" and "*name*, can you keep an eye on the patient’s vital signs a bit while we’re fiddling here?" as well as "Shall we quickly do an ABCDE check now?" These indicate active monitoring and support within the team. | *The example with **ABCDE** I would also place more under **structured communication**.*  **"Alex, can you keep an eye on the patient’s vital signs while we’re fiddling around here?"* — for me, that fits better under **recognizing the importance of teamwork**.* |
|  |  | **Chat-GPT: Structured Communication**  Clear and structured communication can be observed repeatedly:   - "Set the respiratory rate." — The next step is specifically named. - "Then we’ll start induction immediately, that’s my suggestion." — Suggestions are made directly and integrated into the workflow. - "Propofol is running." — Continuous updates on progress provide clarity. | *These examples, in my view, rather illustrate clear suggestions. The structured approach came later with the ABCDE, which I found good. Categorization accuracy issues:* |
|  |  | **Chat-GPT: Structured Communication** Structured communication is evident in phrases like: "Can you go to the head and try to ventilate with the mask?" or "Can you take care of an infusion?" | *These examples, to me, rather indicate Team Mental Models (further below). I recall a started ABC that was interrupted; and later another “Re-Assessment,” which was also announced as such.* |
|  |  | **Chat-GPT: Reflection and Debriefing**  There is little explicit reflection, but the planning demonstrates proactive thinking:   - “Now we need to make a plan first.” (04:16–04:20) - “Let’s say it again together for everyone.” (07:04–07:06) - “That means, now we have to go to the OR.” (13:48–13:50) | *For me, this dialogue was a timeout and gathering of facts (not a debriefing). It would help to know who said each part.* |
|  |  | **Sapient AI: Structured Communication** The communication appears structured, as shown by instructions and feedback such as: "SPEAKER_00: They should come to preoperative." and "SPEAKER_04: Can the blood pressure be triggered every two minutes?" | *From my perspective, these would be examples of clear communication but less so of structured communication. I did not observe the use of specific tools or methods.* |
|  |  | **Chat-GPT: Mutual Performance Monitoring** Team members monitored each other and provided constructive feedback, such as: "But careful, please." "Something’s coming in." "I’ll give another 20." | ***More CLC than performance monitoring*** |
|  |  | **Chat-GPT: Optimization of Shared Mental Models** There are indications of a common understanding, as shown by statements like: "So we simply need to secure the airway," and "We have an airway obstruction, we don’t have a secure airway." | ***This is more Sharing of Information*** |
|  |  | **Chat-GPT: Reflection and Debriefing** There are signs of reflection and debriefing, as shown by statements like: "Now we need to make a plan first," and "So, does everyone understand the plan?" These indicate that the team evaluates the situation and develops a plan together. | ***This is Teamwork not Reflection*** |
|  |  | **Chat-GPT: Creating a Psychologically Safe Environment** There are signs of a psychologically safe environment, such as: "Can you answer the phone, yes, exactly," "Then we need a mask now," and "*name*, I’m just letting him in here," which indicate that team members feel free to speak up and ask for help. | *The above examples, for me, are more like instructions/orders; a psychologically safe environment, in my view, is more about speaking up or asking questions that express uncertainty or point out errors.* |
|  |  | **Chat-GPT: Optimization of Shared Mental Models**  The team works to establish a common understanding:   1. **"We also pulled out the IV catheter."** This information ensures everyone shares the same knowledge. 2. **"The tube was pulled out during positioning."** This provides an overview of the current situation. 3. **"The scope is ready."** This synchronizes expectations. | *I find the distinct separation from other criteria difficult. There was no "10 for 10" or similar — maybe statements from the debriefings?* |
|  |  | **Sapient AI: Asking Clarifying Questions** Clarifying questions are asked to avoid misunderstandings, such as: "SPEAKER_01: But did she respond to you before?" and "SPEAKER_03: Do you have everything ready?" | ***This is sharing of information*** |
|  |  | **Chat-GPT: Mutual Trust**  Trust within the team was evident:   - “Please prepare everything for the cricothyrotomy.” – “Yes.” → Confidence in carrying out the instruction. - “I’m intubating. Will this work?” – “You can do it.” → Encouragement in a critical situation. - “Can you ventilate?” – “Wait, I’m trying.” → Open communication without fear of failure. | *I would definitely consider the second example as appropriate. The other examples are good but, in my view, partly belong more to other categories:*  *The first example is more structured communication plus closed-loop feedback with clear instruction and response (closed loop), but it remains unclear if the task was correctly understood, since the instruction isn’t repeated in the receiver’s own words. The third example I would classify more as clear feedback.* |
|  |  | **Sapient AI: Asking Clarifying Questions** Clarifying questions were asked to avoid misunderstandings: "SPEAKER_00: She has no allergies?" and "SPEAKER_04: Can you hear me?" | *The latter example is more about questions directed at the patient; however, I also recall multiple questions exchanged among each other and directed to the simulation team.* |
|  |  | **Chat-GPT: Optimizing Shared Mental Models**  The team demonstrates a shared understanding:   - "We are now performing a cricothyrotomy." → Clear coordination before a critical intervention. - "We’re checking everything: A, B, C." → The checklist ensures a common understanding of the situation. - "Let’s secure that and then perform a bronchoscopy." → Coordinated approach to planning further actions. | *I find the points good, even though they contain more elements of structured communication.* |
|  | Overgeneralized, Superficial, or Ambiguous Categorization | **Sapient AI: Reflection and Debriefing** SPEAKER_02 reflects on the situation: "We have an airway problem." SPEAKER_02 plans the next steps: "We will ventilate with four hands." SPEAKER_02 summarizes the situation and plans further actions: "We have a secured airway. Now we’ll do a quick 10 for 10." | *These points would be better placed under "Optimization of Shared Mental Models." In themselves, these quotes do not necessarily indicate that reflection has taken place.* |
|  |  | **Sapient AI: Reflection and Debriefing** There are reflections on the experiences and decisions made during the simulation: "SPEAKER_01: I was still quite confused, and then we just... Jogi, tell me, how did the decision to count come about?" | *I can't really categorize these excerpts at the moment; they don’t make enough sense to me right now to be classified as reflection.* |
|  |  | **Sapient AI: Mutual Trust** SPEAKER_00 shows trust in the team by mentioning his return after a break: "It’s nice to see you all again. I’m back after some time off." SPEAKER_04 trusts SPEAKER_02’s instructions: "ORL has been informed; you send it as soon as possible." | *These two points from the category “mutual trust” seem strongly taken out of context to me — the first one completely, and the second is more an element of closed-loop communication and should be categorized there.*  *On a broader level, many good communication points are highlighted, although they are strongly removed from context. Overall, the wording is very assertive and explicit (e.g., “...team members understand their roles and collaborate...”) and, in my view, does not sufficiently question the background of the statements themselves — for example, in the first section from the discussion between Speaker 03 and 04.* |
|  |  | **Chat-GPT: Mutual Trust**  Mutual trust is evident in the collaboration:   - "I’ll give it a try." – "Very good." (16:10–16:13) - "I’ll try it and you handle the conjuncturing." – "Yes, that’s good." (16:03–16:07) - "Try to blink her." (07:22–07:27) – Suggestions are accepted. | *I find it difficult to identify examples for this; I'm also not sure whether the ones listed here are appropriate.* |
|  |  | **Chat-GPT: Mutual Trust**  Trust was demonstrated in:   1. Quote: "Should I still do it?" Response: "Yes, that’s right." (00:14–00:17) 2. Quote: "Franz, I’m really sorry." Response: "It’s okay." (06:14–06:15) 3. Quote: "Breath sounds audible on both sides." (07:54–07:56) ➔ Trust in the colleague’s observations. | *Odd conclusion; how does the AI come to that...?* |
|  |  | **Sapient AI: Closed-Loop Communication** SPEAKER_01 confirms the medication: "100 given after induction." SPEAKER_02 gives instructions for the intubation: "Please prepare the video laryngoscope and the tube on site." SPEAKER_01 confirms the action: "Yes, sir." | *The positive examples for closed-loop communication are well chosen, but still presented too vaguely in depth. For example: "Jawohl" can indicate that the message was heard, but it doesn't necessarily mean the task was understood — as in: "Jawohl, I’m preparing the videolaryngoscope and the tube." Perhaps it would be helpful to initiate a discussion with this input: "Jawohl" suggests that the instruction was acknowledged, but it's not sufficient to determine whether the task was clearly understood without doubt.* |
|  |  | **Chat-GPT: Mutual Performance Monitoring** Team members monitored and supported each other, as in "Please suction." and "Do we have a pulse?". | *An example would be more along the lines of “Can I help you?” or “Shall I take that over for you?” – "Suction, please" would more likely fall under task delegation rather than mutual performance monitoring.* |
|  |  | **Chat-GPT: Closed-Loop** **Communication** Closed communication loops are evident, as shown by statements like "Senior physician continues," "Mask," and "Belt is here," indicating that instructions were received and confirmed. | *I can’t tell from this whether it was a question or part of closed-loop communication.* |
|  |  | **Chat-GPT: Recognizing the Importance of Teamwork** The importance of teamwork was highlighted through coordinated collaboration and clear instructions such as "Can you do that?" and "Can you get a senior physician, please?" The different roles within the team became evident through task allocation and mutual support. | *The different roles in the team became evident through task allocation and mutual support: However, in this scenario, the task distribution was not very clear to me. It would be interesting to know how the AI identifies this.* |
|  |  | **Chat-GPT: Sharing Unique Information** Critical information was shared, such as "The positioning pulled out the tube," "We just gave 20 more sufentanil," and "The tube dislodged due to the positioning." | *These are shared pieces of information. I would not classify them as unique or crucial.* |
|  |  | **Chat-GPT: Sharing Unique Information**  Critical information is shared to create a common understanding:   1. "The tube has been secured again, but everything is so flexible." (00:22). 2. "Now, I think we need to deepen a bit." (02:58). 3. "That means we might be able to reduce a bit again, but for now it’s actually okay." (10:12). | *=> Depending on what was said before statements 2 and 3, they could also be part of a 10-for-10 briefing or a handover.* |
|  |  | **Sapient AI: Mutual Performance Monitoring** SPEAKER_01 monitors medication: "I'm switching from Propofol to gas and will assist with some ventilation." SPEAKER_02 monitors the intubation: "Please prepare the videolaryngoscope and the tube on site."SPEAKER_02 checks the performance of the cricothyrotomy: "Incision, incision like this, horizontal." | *The corresponding quotes seem to me more like actively expressed thoughts. How they are assigned to the topic of "mutual performance monitoring" is difficult for me to understand—probably because more context would help reconstruct the situation more clearly.*  *There is also a general reference to missing points. However, the specific points to which this overall assessment refers are missing here.* |
|  | More Representative Quotes Could Have Been Chosen | **Chat-GPT: Structured Communication** Structured communication was used, for example:   - "Now the peak pressure is at 30" - "We now have slightly increasing ventilation pressures there" - "Now you don’t have to do anything for a moment, please", which indicates clear instructions. | *To me, genuine examples of structured communication would be standardized procedures like ABCDE or 10 for 10. The statements mentioned above do not qualify as such. During the scenario, I did hear and code an ABCDE communication that was clearly different in content. The cited statements come across more as classic “talking to the room” — verbalizations aimed at sharing information without clear structure or targeted communication.* |
|  |  | **Sapient AI: Structured Communication** Team communication appears to be structured, as illustrated by the following quotes:   - "SPEAKER_02: I would now almost suggest we move toward induction." - "SPEAKER_01: Set the respiratory rate." - "SPEAKER_01: Stethoscope, please." | *These examples don’t fit well in my view. Toward the end of the scenario, I did hear a complete ABCDE being used; and during the problem analysis, phrases like “difficult airway” and “Cannot Ventilate Cannot Intubate” were said on the phone.* |
|  |  | **Chat-GPT: Recognizing the Importance of Teamwork** The significance of teamwork became evident in this scenario, particularly through coordinated actions and purposeful communication among team members. An example is the statement: *"Are you ready with the positioning? Side rails on, everything in place?"* This question implies coordination regarding the readiness of preparations and demonstrates that team members are aware of their respective tasks and roles, actively aligning them with one another. | *The above quote comes from the positioning nurse right at the beginning of the scenario; I find it inappropriate because the positioning nurse leaves the scenario after about 2 minutes and does not return. I would find it better if quotes from later parts of the scenario were included.* |
|  |  | **Sapient AI: Structured Communication** SPEAKER_03 provides structured information about the patient: "He has a gastric bypass, that’s what's scheduled next. He is intubated." SPEAKER_01 confirms the size of the venous access: "1.17-gauge." | *SPEAKER_03 provides structured information about the patient: "He has a gastric bypass, which is scheduled next. He is intubated." SPEAKER_01 confirms the size of the venous access: "1.17 gauge."* |
|  |  | **Chat-GPT: Reflection and Debriefing**  There are indications of reflection and adaptation of strategy:   - "That's a good input." — Reflection occurs, and new perspectives are acknowledged. - "Maybe let’s see if we should suction the tube again." — Adjustments are made based on observations. - "Then let’s try to block it." — The team considers how to proceed and adapts to the situation collaboratively. | *"I don’t find these examples fitting for this category. Reflection did not really occur here; at the end, there was an ABCDE assessment, which in my view had a more clearly reflective character."* |
|  |  | **Chat-GPT: Mutual Trust** Mutual trust is established, as seen in statements like "*name* is at the head, with me as backup" and "*name* wants to administer the medication", indicating that roles and responsibilities are clearly assigned and trusted. | *= Role distribution I would not describe this as trust. However, in the debriefing, Greta mentioned that she felt or perceived trust when hearing the statement: ‘Greta, WE need a plan.’"* |
|  |  | **Sapient AI: Asking Clarifying Questions** Clarifying questions were asked to avoid misunderstandings: "SPEAKER_00: She has no allergies?" and "SPEAKER_04: Can you hear me?" | *“The latter example is more directed at questions to the patient; however, I also recall several questions exchanged between team members and to the simulation team.”* |
|  |  | **Sapient AI: Sharing Unique Information** Critical information is shared to improve understanding of the situation: "SPEAKER_08: No CO₂, the patient is cyanotic." | *“I mean the information came from ‘voice to the room’; in my opinion, there were other situations I would have assigned here; e.g., from Lisa at about 54:30: ‘With mask ventilation, he’s rising and falling...’; 55:08: Lisa: ‘Mask ventilation isn’t working…’; 50:36 (don’t know who it was): ‘...he’s still not relaxed…’; 58:32 from the senior physician (OA): ‘I’m not getting anything in… we probably have to cut him open…’; a few seconds later: OA: ‘ONE attempt…’, then nurse: ‘XY is back in…’ — this also fits with the shared mental model.”* |
|  |  | **Sapient AI: Optimization of Shared Mental Models** There are indications of a shared understanding within the team:   - "SPEAKER_00: I’ve now fully lined up, oxygen." - "SPEAKER_04: She is an ORL patient." | *“Now we are thinking about what we will do next… Escalation methods are… The fiberoptic probably won’t help much, it’s already not working… The fiberoptic is ready… We’ll try it once more like this… You do this, and I do that… And if that doesn’t work, maybe the ENT surgeons will be here, who have done this more often than we have.”* |
|  | Desire for More Context or Stronger Exemplification |  |  |
|  |  | **Chat-GPT: Recognizing the Importance of Teamwork** The importance of teamwork was recognized, as illustrated by the statement, "*name*, we need help in OR8 with the patient. Please come quickly from surgery." The interprofessional roles were understood and appreciated, as shown by the request for surgical support. | *Good example, but it refers to help from outside the anesthesia team. Any other examples?* |
|  |  | **Chat-GPT: Recognizing the Importance of Teamwork**  The team recognized the relevance of interprofessional collaboration, as demonstrated by the ongoing coordination. For example, in "I'll get the airway cart." (01:00–01:02) and the assignment of additional tasks such as "Can you get a senior physician, please?" (01:02–01:04), it became evident that all roles were understood and respected. An exceptional example is the proactive coordination to evaluate new options when the endotracheal tube caused difficulties. | *I think the proactive coordination is a good observation. It's a pity that the corresponding dialogue isn't included.* |
|  |  | **Chat-GPT: Mutual Performance Monitoring**  Examples of mutual monitoring include:   1. Quote: "Make sure everything is already in place." (00:31–00:33) 2. Quote: "Arm boards on, everything attached?" (00:22–00:23) 3. Quote: "The saturation is increasing." (07:18–07:24) | *I find it difficult to assess based on just one sentence; I would need more context information for validation.* |
|  |  | **Chat-GPT: Structured Communication**  Structured communication protocols are consistently applied:   1. “We’re seeing slightly increasing ventilation pressures now” (01:20) precisely describes the situation. 2. “With an LMA, the patient is ventilated, we have CO₂, we have a slightly high respiratory rate” (10:01) provides an overview of the patient’s status. 3. “Then relax, absolutely, please” (04:00) is a clear, prioritized instruction. | *I think these statements are parts of structured communication; what was said before and after? More information would help me make a judgment. What is certainly not wrong, though, is that communication is taking place.* |
|  |  | **Chat-GPT: Structured Communication**   - “We need—*name*, can we get someone else to help…?” (05:22) shows the willingness to involve external support. - Despite the stressful situation, the team strives to act in a coordinated manner, for example through clearly defined responsibilities such as securing the airway and administering medication. | *Yeah. The tasks were assigned. However, the related quotes are missing here.* |
|  |  | **Sapient AI: Optimizing Shared Mental Models** There is evidence of a shared understanding within the team, as demonstrated by the discussion about the need to intubate the patient: "SPEAKER_07: Mask ventilation isn’t working." | *There is also a general reference to missing points. However, the specific points on which this overall assessment [which in some areas could benefit from clearer communication patterns] is based are missing here, as explicit examples are lacking.* |
|  |  | **Sapient AI: Mutual Performance Monitoring** SPEAKER_01 monitors medication: “I’m switching from propofol to gas and will provide some assisted ventilation.” SPEAKER_02 monitors the intubation: “Can you please get the video laryngoscope and have the tube ready.” SPEAKER_02 checks the execution of the cricothyrotomy: “Incision, incision like this, horizontal.” | *The corresponding quotes seem to me more like actively expressed thoughts. How these are assigned to the topic of “Mutual Performance Monitoring” is difficult for me to understand—probably because a bit more context would help reconstruct a clearer situation.* |
| **Identification of Problems** | Misidentification of Speaker or Role | **Chat-GPT: Asking Clarifying Questions** Clarifying questions were asked to avoid misunderstandings, such as “Can you hear me?” and “What do I hear? I hear very poorly. Is that correct?” | ***These are questions for the VOG*** |
|  |  | **Chat-GPT: Creating a Psychologically Safe Environment**  There are signs that team members communicate openly and feel safe:   1. “Are you pressing on something right now, or?” – “No?” (01:10–01:14) shows that questions can be asked without fear of consequences. 2. “I don’t know why I’m there…” – “Am I already in?” (12:52–13:04) shows that uncertainties can be expressed. 3. “What’s alarming again now?” – “Do we need to pay attention to this alarm?” (11:59–12:08) shows that questions are accepted and answered. | ***These are questions for the VOG*** |
|  |  | **Chat-GPT: Recognizing the Importance of Teamwork** The importance of teamwork was recognized in this scenario, as demonstrated by the coordinated actions and communication among team members. An example of this is the discussion about attaching the arm boards and preparing for positioning: “Are you ready with positioning? Arm boards on, everything attached?” This shows that the different roles and tasks within the team were understood and carried out. | *So-so ... I mean, this question came from the ESP (Embedded Simulated Person), not from a team member. I would like to see some other examples here.* |
|  |  | **Chat-GPT: Creating a Psychologically Safe Environment** There are indications that a psychologically safe environment was established. Team members express concerns and ask for help, as in the situation where a mistake occurred: “Oh no, I slipped there. I pulled out the line and the tube. *name*, I’m really sorry. Did I do something wrong? What should I do now?” | *This question also came from the ESP (Embedded Simulated Person), not from a team member. Therefore, the conclusion here—based on the given example—is not correct. I would like to see some other examples here.* |
|  |  | **Chat-GPT: Structured Communication** The communication appears to be structured, with clear instructions and questions asked to check the patient’s condition and coordinate the next steps: “Patient is blue. FOP control center. Hi, *name*, what’s your name?” | *"I did not find the communication very structured; I missed an ABCD approach or an explicit initiation of a time-out / 10 for 10. In my opinion, the examples mentioned above also came from the simulation colleagues, in this case through the ‘voice in the room.’"* |
|  |  | **Sapient AI: Asking Clarifying Questions** Clarifying questions are asked to ensure that all team members are on the same page: “SPEAKER_06: How much time do you have left? SPEAKER_06: 5 minutes.” | *"I mean, the question ‘How much longer do you have?’ also came from outside the team and probably shouldn’t be included here. We would somehow need to mark speakers and be able to exclude them from the interpretation, e.g., instructors, ESPs…"* |
|  |  | **Sapient AI: Sharing Unique Information** Critical information is shared to improve understanding of the situation: “SPEAKER_08: No CO₂, patient is blue.” | *"I mean, this information also came from outside the team and probably should not be included here. We would need to somehow mark speakers and be able to exclude them from the interpretation, e.g., instructors, ESPs..."* |
|  |  | **Sapient AI: Optimizing Shared Mental Models** There are indications of a shared understanding within the team, as shown by the discussion about the need to intubate the patient: "SPEAKER_07: Mask ventilation isn’t working." | *Depends. If Speaker_07 is a team member, then it’s a great example; if they are external (instructor speaking into the room), then we shouldn’t count it...”* |
|  |  | **Sapient AI: Mutual Performance Monitoring** Team members monitor each other and provide feedback, as seen in the discussion about the correct placement of equipment: "SPEAKER_06: Make sure everything is already in place." | *I think we also need to ensure that only things from inside the scenario room are recorded, or arrange for a voice in the control room.”*  *“What else I noticed: It would be interesting to see if the ‘voice to the room’ could be identified; often, patient-relevant information is communicated through it, which the AI mistakenly assigns to the team’s communication flow.”* |
|  |  | **Sapient AI: Mutual Trust** Mutual trust is built through support and understanding of mistakes: "SPEAKER_00: Franz, I’m really sorry. Did I do something wrong now?" | *** Comes from the positioning nurse right at the beginning of the scenario, in my opinion doesn’t fit here because it is scripted behavior.”* |
|  |  | **Chat-GPT: Sharing Unique Information** Some team members share important information that others do not have:   1. “At the moment, we still have 84% saturation.” (02:48–02:51) 2. “There is bleeding in the mouth.” (05:11–05:16) 3. “The tumor is very, very large.” (04:48–04:56) This information is essential to accurately assess the situation. | ***I think quote 1 and 2 are not from the Sim Team*** |
|  |  | **Chat-GPT: Mutual Trust**  Trust was demonstrated in:   1. Quote: “Should I still do it?” Response: “Yes, that’s right.” (00:14–00:17) 2. Quote: “*name*, I’m really sorry.” Response: “It’s okay.” (06:14–06:15) 3. Quote: “Breath sounds audible on both sides.” (07:54–07:56) ➔ Trust in the colleague’s observations. | *** Inappropriate, see comment on the right; my observation: around 55:44: OA arrives, AA gives a brief handover, nurse asks question(s). I meant this comment came from the voice to the room.* |
|  |  | **Chat-GPT: Structured Communication** The communication appears to be structured, with clear instructions and questions asked to assess the patient’s condition and coordinate the next steps: “Patient is blue. FOP control center. Hi, *name*, what’s your name?” | *** I meant that was said by the positioning nurse at the beginning; scripted, I find it inappropriate; otherwise, I didn’t hear any closed-loop communication.* |
|  |  | **Sapient AI: Closed-Loop Communication** There are examples of closed-loop communication, where instructions are confirmed and carried out: “SPEAKER_07: The arm board on the left is still missing. SPEAKER_07: The right one is missing too. SPEAKER_07: Should I still put it on? SPEAKER_07: Yes, that’s correct.” | ** I mean the info came from the voice to the room; in my opinion, there were other situations I would have assigned here; e.g. from Lisa, around 54:30: “Mit Maske beatmen, hebt und senkt, …”; 55:08 Lisa: “Maskenbeatmung geht nicht…”; 50:36 (don’t know who that was): “…relaxiert ist er noch nicht…”; 58:32 from OA: “Bekomme gar nichts rein…, müssen ihn wohl aufschneiden…”; a few seconds later: OA: “EIN Versuch…” then nurse: “XY ist wieder drin..” => also fits with shared mental model* |
|  |  | **Sapient AI: Sharing Unique Information** Critical information is shared to enhance understanding of the situation: "SPEAKER_08: No CO₂, patient is blue." | *The above quote is from the positioning nurse at the very beginning of the scenario. I find it inappropriate because the positioning nurse leaves the scenario after about two minutes and does not return. It would be better to include quotes from later in the scenario or to supplement this quote with statements from the later course of the scenario.* |
|  |  | **Chat-GPT: Recognizing the Importance of Teamwork** The importance of teamwork was recognized in this scenario, as demonstrated by the coordinated actions and communication among team members. An example of this is the discussion about attaching the arm boards and preparing for positioning: “Are you ready with positioning? Arm boards on, everything attached?” This shows that the different roles and tasks within the team were understood and carried out. | ***Quote 2 is from an instructor.*** |
|  |  | **Chat-GPT: Sharing Unique Information**  Critical information is shared effectively:   - “She is an ORL patient.” (05:29–05:31) – shares the patient background with the team. - “You only see bloody, slightly bleeding tumor tissue.” (11:28–11:30) – observations are directly communicated. - “Base of tongue carcinoma, planned.” (02:52–02:56) – highlights context for planning. | ***Quotes 1 and 2 are from instructors.*** |
|  |  | **Chat-GPT: Mutual Trust**  Trust was demonstrated in:   1. Quote: “Should I still put it on?” Response: “Yes, that’s right.” (00:14–00:17) 2. Quote: “*name*, I’m really sorry.” Response: “It’s okay.” (06:14–06:15) 3. Quote: “Breath sounds audible on both sides.” (07:54–07:56) ➔ Trust in the colleague’s perception. | *I think these remarks also came from people outside the actual team — is that usable/relevant?* |
|  |  | **Chat-GPT: Sharing Unique Information**  Important information was shared:   1. Quote: "Patient is blue." (01:06–01:38) 2. Quote: "No CO₂, patient is blue." (01:49–02:05) 3. Quote: "The neck is short and thick, nothing can be felt from the outside." (06:04–06:13) | *I’m no longer sure whether these quotes came from the instructor team, the surgeon via phone, or the ESP (positioning nurse).* |
|  |  | **Chat-GPT: Asking Clarifying Questions**  Clarifying questions were used to avoid misunderstandings:   1. Quote: "Are you ready with positioning?" (00:19–00:22) 2. Quote: "Did I do something wrong now?" (00:56–00:58) 3. Quote: "How much time do you have left?" (00:34–00:36) | *Quotes 1 and 2 came from the ESP and should not count here; quote three from whom? → I notice that here no different Speaker_0X labels are marked….* |
|  |  | **Chat-GPT: Recognizing the Importance of Teamwork** The transcript shows that teamwork was overall recognized as central. In particular, the exchange of tasks and the effort to keep each other informed is evident:   - Quote: "Make sure everything is already in place. Then I can start washing up." (00:31–00:34) ➔ This indicates an awareness of the necessary preparation by all team members. - Quote: "Are you ready with positioning?" (00:19–00:22) ➔ Here, it is asked whether everyone is ready, emphasizing the importance of interdisciplinary collaboration. | *I think quote 3 is also from an instructor. Overall, I perceived little structured communication.* |
|  |  | **Chat-GPT: Creating a Psychologically Safe Environment**  Signs of a psychologically safe environment:   1. Quote: "*name*, I’m really sorry." (00:54–00:56) ➔ The person openly admits a mistake and apologizes. 2. Quote: "Did I do something wrong now? What should I do?" (00:56–00:59) ➔ Questions indicating uncertainty are asked. 3. Quote: "Oh, come on. Sorry." (06:13–06:14) ➔ This shows willingness to acknowledge one’s own errors. | ***These quotes are not from the Sim Team*** |
|  |  | **Chat-GPT: Structured Communication**  There were attempts to give clear instructions, but communication could have been more structured:   1. Quote: "Make sure everything is already in place." (00:31–00:33) 2. Quote: "The arm boards on the left are still missing." (00:12–00:14) 3. Quote: "Tongue. No epiglottis, no vocal cords." (04:23–04:24) ➔ Clearly formulated observations | *Quote 1, in my opinion, is from the ESP — therefore not usable here. Quotes 2 and 3, in my opinion, are from the control room — so probably not usable here either, right?* |
|  |  | **Chat-GPT: Reflection and Debriefing**  There is little evidence of structured reflection, however:   1. Quote: "Did I do something wrong now? What should I do?" (00:56–00:59) ➔ Reflection on one’s own actions. | ***No reflection.*** |
|  |  | **Chat-GPT: Reflection and Debriefing**  There is little evidence of structured reflection; however:   1. Quote: "Did I do something wrong now? What should I do?" (00:56–00:59) ➔ Reflection on one’s own actions. 2. Quote: "It doesn’t get better with that word." (09:12–09:13) ➔ Honest assessment of the situation. 3. Quote: "How did the decision come about to count?" (08:32–08:34) ➔ Subsequent clarification. | *In my opinion, this comes from the positioning nurse at the beginning; it is scripted and I would exclude it.* |
|  |  | **Chat-GPT: Structured Communication** Structured communication was used, as seen in statements like "Now he is at a peak pressure of 30," "We now have slightly increasing ventilation pressures," and "Now you don’t have to do anything for a moment, please," which indicate clear instructions. | *For me, structured communication would be an ABCDE assessment or a “10 for 10” check; in my opinion, the above statements are not examples of that. During the scenario, I heard and coded an ABCDE assessment that was different in content. The statements above seem to me like classic “talking to the room,” verbalizations into the room (just sharing information).* |
|  |  | **Chat-GPT: Structured Communication**   1. Quote: "Make sure everything is already in place." (00:31–00:33) 2. Quote: "The arm boards on the left are still missing." (00:12–00:14) 3. **Quote:** "Tongue. No epiglottis, no vocal cords." (04:23–04:24) ➔ Clearly stated observations. | *Those don’t sound like questions to me. It sounds like information coming from the voice to the room.* |
|  |  | **Chat-GPT: Closed-Loop Communication**  Examples of closed-loop communication:   1. Quote: "Arm boards on, everything on?" (00:22–00:23) ➔ Confirmation follows: "Yes, that’s correct." (00:15–00:17) 2. Quote: "CO2, CO2, CO2." ➔ Response: "The saturation is rising." (07:18–07:24) 3. Quote: "Breath sounds audible on both sides." (07:54–07:56) ➔ Demonstrates successful confirmation of the observation. | *I don’t recall that this information came from the team; it sounds more like information from the voice to the room.* |
|  |  | **Chat-GPT: Sharing Unique Information** Crucial information was shared, such as "The patient does not respond to waking and snapping out," and "The tumor is very, very large." | *In my opinion, these examples came from the instructors via the voice to the room. I observed more questions rather than information sharing.* |
|  |  | **Sapient AI: Reflection and Debriefing** There are reflections on the experience, such as: "SPEAKER_04: Thank you very much for the scenario." and "SPEAKER_02: Let's leave it like this now before I break the doll, okay?" | *The examples do not fit because they came from the instructors – I did not perceive any reflection or subsequent joint evaluations.* |
|  |  | **Closed-Loop Communication** Messages were correctly received and confirmed, as shown by statements like "The scenario continues," "Okay," and "Intubation is ready." | *Good examples, I hadn’t noticed them at all. “Scenario continues” — from the instructor...* |
|  | Misinterpretation of Technical Terms or Abbreviations | **Sapient AI: Sharing Unique Information** SPEAKER_03 shares specific patient details: “He has a 17-gauge eyelash, lying down there.” SPEAKER_01 provides medication details: “They gave 0.2 mg fentanyl, 100 mg Esmeron, and the propofol is running at 4.” SPEAKER_02 shares the plan for emergency intervention: “We now need help from the URL, and we are going to perform a cricothyrotomy.” | *That the technical terms were not correctly recognized by either of them does not bother me much. I was able to recall what was actually said each time. Once, even both AIs quoted it incorrectly in the same way ; Misinterpretation of technical terms or abbreviations*  *Overall, the report is clearly designed and well structured. The translation—especially of technical terms or abbreviations—is difficult and still contains some gaps.* |
|  |  | **Chat-GPT: Sharing Unique Information** Critical information was shared, such as “This is Mr. Rutschmann, who was intubated during a jump,” “He was given a 4-dose of propofol,” and “The positioning nurse just now—although we were actually already ready—pulled out the tube during positioning,” which shows that important information was communicated within the team. | *The translation of specialized terms is somewhat lacking here, but important core aspects were conveyed. In these examples, I also perceive some overlap with the topic of “structured communication.” The first two quotes are a bit short, so they don’t allow me to clearly infer the context without doubt. The last quote from Speaker02 contains valuable context, which I find helpful.*  *I coded it as a handover from AA to OA after the incident with the positioning nurse; what does "4-wattig" mean...?* |
|  | Overconfident or Unwarranted Wording by AI | **Chat-GPT: Optimization of Shared Mental Models**  The team demonstrates a shared understanding:   - "We are now performing a cricothyrotomy." → Clear coordination before a critical intervention. - "We’re checking everything: A, B, C." → The checklist ensures a common understanding of the situation. - "Let’s fix this and then perform a bronchoscopy." → Coordinated approach to planning further actions. | *I find these points good, even though they contain more elements of structured communication. What is somewhat problematic is the statement that these individual events indicate a good shared mental model, because during the debriefing it became clear that this mental model, at least at the beginning, was different and had to be aligned first.* |
|  |  | **Chat-GPT: Mutual Trust**  There are several signs of mutual trust:   1. “*name*, I’m really sorry.” (00:54–00:56) – The apology indicates trust in the relationship. 2. “Did I do something wrong now?” (00:56–00:58) – Uncertainties are openly expressed. 3. “That’s fine.” – “That’s fine.” (06:14–06:15) – Mutual confirmation. Trust appears to be situational but could be further strengthened through more structured communication. | *On a larger scale, numerous good communication points are highlighted, although often strongly taken out of context. Overall, the wording is very assertive and explicit (“…team members understand their roles and collaborate…”), and in my view, it insufficiently questions the underlying context of the statement itself, such as in the first paragraph drawn from the discussion between Speaker 03 and 04.* |
|  |  | **Chat-GPT: Mutual Trust**  Trust was demonstrated in:   1. Quote: "Should I still put it on?" Response: "Yes, that’s correct." (00:14-00:17) 2. Quote: "*name*, I’m really sorry." Response: "That’s fine." (06:14-06:15) 3. Quote: "Breath sounds audible on both sides." (07:54-07:56) ➔ Trust in the colleague’s observations. | *The interpretation of quote 3 is interesting… it must be a repetition of what the person who was listening said. How did the AI recognize that?!* |
|  | Systematic Biases in AI Interpretation | **Chat-GPT: Recognition of the Importance of Teamwork**  The team demonstrates a strong awareness of the importance of collaboration, especially in emergency situations:   - "Who is at the head?" – "I am at the head." → The clear role assignment shows that team members know and carry out their responsibilities. - "Are you monitoring circulation and anesthesia?" → This statement highlights the importance of interprofessional cooperation, with anesthesia and nursing working closely together**.** | *I think these are nice examples of teamwork. In the first example, "Who’s at the head? – Me," the overlap with the topic of closed-loop communication is very large and it could also fit well there or as a great combination of two commonly discussed themes (closed loop AND role distribution). The second example fits perfectly for task allocation. However, I wonder what knowledge this statement is based on that this happened specifically between doctor and nursing staff. Could there be a bias at play, where different professions are automatically assigned corresponding role distributions? (For example, the assumption that the doctor must be in the lead and nursing staff certainly cannot be?!)* |
|  |  | **Chat-GPT: Structured Communication** The communication appears structured, with clear instructions and questions aimed at assessing the patient’s condition and coordinating the next steps: "Patient is blue. FOP control center. Hi, *name*, what is your name?" | *I didn’t find the communication very structured; I missed an ABCD approach or an explicit initiation of a time-out / 10-for-10. In my opinion, the examples mentioned above also came from the simulation colleagues, in this case through the “voice to the room.”* |
|  |  | **Chat-GPT: Structured Communication**  There were attempts to give clear instructions; however, the communication could have been more structured:   1. **Quote:** "Make sure everything is already prepared." (00:31-00:33) 2. **Quote:** "The handrails on the left are still missing." (00:12-00:14) 3. **Quote:** "Tongue. No epiglottis, no vocal cords." (04:23-04:24) ➔ Clearly formulated observations. | *It would be interesting if, in addition, central points that were missing could be proposed. (e.g., missing coordination, lack of closed-loop communication, or use of destructive language)*  *I think Quote 3 is also from an instructor. Overall, I perceived little structured communication.* |
|  |  | **Chat-GPT: Closed-Loop Communication**  Messages were correctly received and confirmed:   - "Please call the senior physician." – "I am calling the senior physician." → Clear command that is executed and acknowledged. - "Can we ventilate?" – "No, it’s not working." → Precise feedback to critical questions. - "Do we have CO2?" – "CO2 is coming." → The response confirms the successful action. | *Nice examples, also very aptly described. I like that a lot! What I find interesting here is whether missing feedback (i.e., absent closed-loops) is also considered a topic in the transcript analysis? I personally did not notice any missing closed-loops, but if this were also an observation point, it would definitely support the debriefers in reducing their “blind spots.”* |
| **Suggestions for Improvement** | Link Quotes to Timestamps and Identify Speakers | **General comments** | *What was not captured: who is leading? This often arises in reality from the context, which the AI cannot recognize at all. In general, I miss the timestamps and the assignment to persons here.*  *Important for me in both cases would be an assignment to persons. And the time indication is important.*  *I am missing a timestamp. This would help me to assess whether these quotes stand alone or were possibly said during a timeout. The timestamp would also help me find the video clip or recall the situation. Then I could deduce what led to the quote and what effect it had.* |
|  |  | **Chat-GPT: Closed-Loop Communication** There were closed communication loops, such as "Then put to sleep, had 0.2 Fentanyl and 100 Esmeron" and "Then we need a mask now" as well as "And please, maybe you could call our senior physician," which shows that messages were received and confirmed. Yes. These sentences were said. I would rather assign them to sharing information. It would help me here if the quotes were assigned to persons. | *I miss the names of the speakers here. I find timestamps extremely helpful to put the context in perspective and possibly show the video clip. That would also help here to see what led to the statement and what effect it had or if it was noticed at all.* |
|  |  | **Chat-GPT: Closed-Loop Communication**  Messages were correctly acknowledged and executed:   1. "*name*, can you check if the positioning nurse really called *name*?" – "That's the one." (03:30–03:39) 2. "Draw up adrenaline once?" – "Here, just draw this up." (06:30–06:44) 3. "I need a bit more." – "Yes, I think it’s not going in." (04:17–04:21) Yes, these dialogues were observable. It would be good if they were assigned to persons here. | *What is missing are assignments to persons. For example, it is not possible to tell from the transcript whether one person is speaking multiple sentences or if it is a dialogue.* |
|  |  | **Chat-GPT: Optimizing Shared Mental Models**  The team creates a shared understanding of the situation:   1. "With a LAMA, we are ventilated, we have CO2, we have a bit of a high ventilation frequency at the moment." (10:01) 2. "Now we have 15, right?" – "So maybe it really was some form of bronchospasm reaction." (10:30–10:41) 3. "That means we might be able to go down a bit again, but now it’s basically okay." (10:12) This was during a 10-for-10 (timeout). It would help me if the quotes were assigned to persons. | *I am wondering whether it is possible for the AI to recognize this only by the wording. What I really miss here is that the quotes are not assigned to persons.* |
|  |  | **Sapient AI: Sharing Unique Information** Critical information is shared to improve understanding of the situation: "SPEAKER_08: No CO2, patient is blue." I think this info also came from outside the team and perhaps should not be included here. We would need a way to mark speakers and exclude from interpretation, e.g., instructors, ESPs... |  |
|  |  | **Chat-GPT: Closed-Loop Communication**  Examples of closed-loop communication:   1. Quote: "Hand benches on, all on?" (00:22–00:23) → Confirmation follows: "Yes, that’s right." (00:15–00:17) 2. Quote: "CO2, CO2, CO2." → Response: "Saturation is rising." (07:18–07:24) 3. Quote: "Breath sounds audible on both sides." (07:54–07:56) → Shows successful confirmation of observation. | *For me, it would be important to be able to recognize whether statements come from different persons, e.g., the third quote – if it’s from the control room into the scenario room, then it’s not useful and says nothing about the team’s behavior.* |
|  |  | **No category assigned**  The transcript shows clear efforts toward team communication and coordination. Areas for improvement include structuring communication and more comprehensive reflection. Psychological safety within the team appears to be given, as errors were openly addressed and questions asked. | *I miss the timestamps here.* |
|  |  | **No category assigned**  This report offers a detailed analysis of communication and supports a well-founded debriefing. If further details are desired, I am happy to assist. | *I find this report clearly more helpful and concrete compared to the other; in particular, the time sections are very helpful for debriefing. Some example sentences are a bit too short or lack enough context for me to make a valid statement (e.g., "okay, good"). Another positive point in this report: there are more examples, while the other report keeps repeating the same examples.* |
|  | Expand Beyond Text-Based Analysis | **Sapient AI: Creating a Psychologically Safe Environment** SPEAKER_00 feels safe enough to admit being new on the job: "I am new on the job." SPEAKER_01 asks for help: "Can you please call the senior physician?" and SPEAKER_00 admits a mistake: "I am a clumsy man." | *These points are nicely highlighted, although in this case mentioned by the ESP. What is missing here is the depiction of tone of voice to convey the calm and controlled aspect of psychological safety. This is a dimension that largely happens “between the lines” and non-verbally, and it is therefore unsurprising that it is not well represented. As instructors, we pay particular attention to this.*  *The participants did acknowledge that psychological safety was present in the scenario. They attributed this, for example, to the fact that they were standing facing each other. However, the software cannot recognize this.* |
|  |  | **Chat-GPT: Mutual Performance Monitoring**  Team members monitor and support each other:   - “Thorax not moving.” – “Let’s switch to the laryngeal mask.” (10:30–10:36) – Feedback leads to adjustments. - “I hope I don’t break anything on this bump.” – “Well, there is the membrane.” (17:01–17:02). - “Situation keeps dropping.” – “Press the timer.” (10:16–10:20). | *From my perspective, the anesthesia nursing staff observed the other team members very attentively. → Nonverbal – not representable here – potential AI bias*  *What else occurs to me is whether the AI could infer safety feelings or stress levels from speech melody or changes in the pitch of individual persons’ voices. That would be an interesting observation. I can also imagine the AI distinguishing the spoken volume of individual participants over time, e.g., when someone speaks loudly or softly and what influence that has on the team*  *What I could also imagine would be: Do participants talk over each other or one after the other (this could also be graphically displayed using bars, for example)? What is the general volume level and does that influence performance? Or: does the volume decrease after a timeout or something similar?.* |
|  | Improve Result Presentation and Clarity | **General comments** | *I find it very positive that responses to statements are given multiple times. As a master point, I could imagine these quotes with timestamps being directly inserted into our Team First debriefing event list.*  *That would still help me. For the presentation of the transcript, I could imagine the AI transferring the quotes directly into our debriefing or scenario protocol. Alternatively, the quotes could be listed chronologically and color-coded according to whether the AI assigns them to challenges, communication skills, or coordination skills.* |

**Supplementary Table S3:** Categories and complete quotes from Simulation participants. The table presents key themes and illustrative comments from the simulation regarding the advantages, disadvantages, and feedback of AI-supported simulation.

| **Categories** | **Comments** |
| --- | --- |
| **Optimism towards AI technologies** | “Will hopefully be standard equipment in 2 years”  “Can only be advantageous, I think”  “Generally interesting, I work in AI science myself”  “Sees the potential of AI even if he doesn’t know much about it himself”  “Useful, right, reduces workload, shapes the future”  “Fan of AI”  “Open towards AI”  “Exciting — AI is something I can use. Suggestions, an exciting additional resource”  “One should be positive and give AI a try”  “I’m totally open”  “AI is the future — accept and embrace it”  “Thinks Visual Patient with AI features could be good”  “Great”  “A computer can access far more data — something a human cannot do”  “Our human horizon is still limited — we can’t yet imagine what AI will be capable of someday”  “Believes AI can still be a good additional feature”  “I believe useful benefits and help can come from it”  “I think AI should be used meaningfully”  “AI still interesting”  “Very open. It would be foolish to resist it”  “Belief in AI technology”  “New positive aspects should be explored. One should not reject AI as a concept outright”  “Open towards AI”  “AI is the future, definitely. You have to be part of it, or you’ll miss the trend. It’s a race—if you miss the jump, you're left behind”  “It will be the future”  (specific technology mentioned) “A great AI example: the Hamilton ventilator — detects increased airway pressures, anticipates bronchospasm, provides therapy suggestions”  “Believes that in a few years we will have AI assistance at all levels” |
| **Trust in AI technologies** | “Don’t feel observed or affected in my work”  “I have nothing against AI observation”  “I have no concerns”  “Didn’t feel observed, nobody finds it interesting, no one could take anything from it”  “That AI is listening and creating a transcript doesn’t change how I feel”  “AI in simulation — during the scenario, I forgot that AI was running in the background, which can be interpreted as acceptance”  “I don’t see any risks”  “No concerns about data protection with AI”  “Its use doesn’t bother me”  “No burden”  “The earlier you learn to work with it, the more influence you can have AI is inevitably part of the future” |
| **Perceived benefits: Enhanced perception through AI** | “More input as a result”  “An additional aid to humans, because it notices more”  “As humans, we have limited perception and cannot see or hear everything. AI can therefore possibly address other things”  “Believes that AI can better recognize correlations”  “AI can also provide interpretation — sometimes trash, sometimes a new perspective or tunnel vision. Both can be useful”  “Thinks that AI will help with understanding correlations” |
| **Perceived benefits: Independence from human factors** | “Humans make mistakes”  “Open to it, because computer systems are already more accurate and precise”  “Fewer sources of error. Reduces human errors”  “Humans wait for errors from the simulators, based on previous groups / personal experiences”  “Thinks AI is superior to humans in terms of prediction and precision”  “Basically doesn’t miss anything — although we might miss certain things”  “A human cannot be fully objective; AI is objective. AI just looks at what is given and puts it on paper. That can be both an advantage and a disadvantage” |
| **Perceived benefits: Objectivity and impartiality** | “AI is impartial, impersonal, evaluates information repeatedly”  “Unlike human evaluation, prevents bias from previous errors that have occurred in the past”  “AI has no emotions, makes it non-judgmental, has no memory, doesn’t judge”  “Like a hyper-intelligent autistic person”  “AI can increase quality and safety — it’s an additional safety tool”  “Has no emotions, which is an advantage — an emotionless evaluation. AI is purely factual, whereas humans come with experiences and evaluate things emotionally. AI doesn’t do that.”  “Where are the priorities? AI should monitor that and take over without bias”  “Computers remove emotions, which may make them fairer in terms of evaluation”  “The machine points out human errors more relentlessly. I like that.”  “Ensures and improves quality”  “AI tells you: that was bad, this is good — that’s what I want”  “I like the simple, unfiltered feedback from the AI”  “Great, because AI can recognize and analyze typical changes” |
| **Distrust towards AI technologies: Perceived risks due to lack of transparency** | “Doesn’t know the backend well, carries the risk of being manipulated”  “Humans remain in the dark regarding the background, gives an uneasy feeling”  “The tendency is to think the computer must know better — but we need to think critically about whether that’s really the case”  “How do you verify the information or recognize if AI has made an error?”  “What does the AI register, and how does it interpret the information?”  “But it can also present facts in a way that makes them seem true. With a human, you notice uncertainty or emotion. A computer appears more confident — but is the info correct?”  “Would like to know who reads the transcript and have influence over who receives it. That would improve things” |
| **Suggestions for important AI features: Support for human situational awareness** | “Ease of visualization will be key”  “I want feedback to see whether AI has understood the purpose”  “Use it in a differentiated way. Evaluation — will we get to see that? Also, something that benefits everyone and is transparent” |
| **Distrust towards AI technologies: Perceived risks due to language barriers** | “Does it understand Swiss German?”  “Can AI understand all dialects and languages?” |
| **Distrust towards AI technologies: Data privacy and security** | “I assume that my face and voice won’t be copied (data protection)”  “Torn about data privacy: Where does the data go? How is it processed?”  “In terms of data protection: I’d rather not have my voice uploaded to AI, but if only transcripts are generated, that’s okay” |
| **Perceived benefits: Support for creative processes and implementation of ideas** | “We have the ideas, and AI helps to realize them”  “The program can also provide good insights”  “Can provide interesting input and be helpful, especially when comparing things”  “Can provide input; AI can help shape further development”  “Good associations, sometimes amusing” |
| **Perceived benefits: Increased efficiency through automation** | “If personal data security is guaranteed, AI can only help us in research”  “Efficiency gains through AI. Data can be better captured and analyzed, faster”  “Can help us summarize information”  “AI use case in obstetrics: triaging patients”  "AI technology can take a lot of work off our hands in the future."  “Outpatient consultation transcripts document ward rounds” |
| **Dismissive attitude towards AI technologies** | “Not a fan of AI” |
| **Distrust towards AI technologies: Perceived risks to cognitive abilities** | “We're becoming dumb because of it, no longer using our own brains”  “Fear of neglecting one's own intuition”  “We just type it into the GPS. There’s no need to remember anything” |
| **Distrust towards AI technologies: Perceived risks to social and communicative skills** | “No need for communication skills anymore, AI limits verbal communication abilities” |
| **Suggestions for important AI features: Data protection and anonymization** | “Should be anonymized, and I prefer anonymized analysis”  “Information on where the data goes — companies? Conflicts of interest?” |
| **Limited experience with AI** | “I'm not familiar with it yet and don’t have many reference points; I know AI from colleagues/acquaintances”  “Not quite used to it yet"  “Currently still learning more about AI”  “I can’t really picture what that would be like”  “Personally used it little” |
| **Distrust towards AI technologies: Perceived risks due to technological errors and limitations** | “More concerned about technological errors”  “How do you verify the information or recognize if AI has made an error?”  “It may be that AI lacks understanding. AI certainly cannot empathize with humans as well as a human can.”  “Disadvantage: it cannot recognize emotions”  “Felt moderately comfortable, a bit stressed”  “Lack of emotion is also a disadvantage, because certain aspects may be missed”  “A simple example is ECG interpretation, which is often incorrect and should be critically evaluated”  “Relying too heavily on it is a problem. If humans no longer carry primary responsibility, it could be problematic”  “It’s important that AI tools are well-trained — that helps with acceptance”  “Not ideal to be misunderstood by AI. I don’t want to be misunderstood” |
| **Impairment due to perceived observation situation** | “Also inhibited by being observed — you don’t feel as free to act, the fact of being observed causes tension, whether by AI or a human” |
| **Neutral towards AI** | “Pretty indifferent”  “Torn / conflicted”  “AI needs to be tested in many areas of medicine, but it’s certainly not a cure-all everywhere” |
| **Trust towards AI technologies: No data privacy concerns** | “No real problem with how it’s used”  “Doesn’t bother me at all” |
| **Criticism of AI’s resource consumption / Sustainability concerns regarding AI** | Energy consumption of computers is an issue — where does the electricity come from? Do we really need it? Responsible use of AI and the environment |
| **Desire for human co-assessment** | “The classification and debriefing should be human-led”  “I’d like to compare the AI’s debrief result with the human debrief result”  “If only AI were to analyze, the human aspect would be missing”  “AI is only as good as the data you feed it. The output must be assessed with expert knowledge. A layperson cannot evaluate the AI output. Both input and output must be compared with human knowledge.” |
| **Perceived benefits: Support in scientific work** | “I see AI in the medical-scientific context as a resource that must be used” |
