# Supplementary material for: Generative AI in simulation debriefings: an exploratory study using the Team-FIRST framework and qualitative feedback from simulation experts and learners
Source: Adv Simul (Lond). 2026 Jan 29;11:14. doi: 10.1186/s41077-026-00407-0 (PMC12924402; doi:10.1186/s41077-026-00407-0)
Supplement: Supplementary file 2 — Supplementary Material 2 [file 41077_2026_407_MOESM2_ESM.pdf]

Supplementary Material to: ***Generative AI in Simulation Debriefings: An Exploratory Study Using the Team-FIRST Framework***

Input (audio transcript by Sapient AI)

**00:00 \~ 00:02 Allergy to fist dust and pollen.
00:02 \~ 00:05 He has a gastric bypass, which is scheduled for surgery next.
00:05 \~ 00:07 He is intubated.
00:07 \~ 00:09 Has he had prior surgery?
00:09 \~ 00:11 No prior surgery, no.
00:11 \~ 00:14 He has a 17-gauge IV access, lying down there.
00:14 \~ 00:15 That is on the arm.
00:15 \~ 00:16 And I extended it here.
00:16 \~ 00:18 He could get injections here.
00:18 \~ 00:19 And so, the line runs down there.
00:19 \~ 00:20 It is like this.
00:22 \~ 00:25 And the arms are tucked in.
00:25 \~ 00:25 Exactly.
00:27 \~ 00:32 It is already disinfected and can start at any moment.
00:34 \~ 00:38 For induction, but he was awake fiber-optically.
00:38 \~ 00:44 He was given 0.2 fentanyl, 100 Roc., and the propofol is running at 4 TCI.
00:47 \~ 00:51 And he is asleep.
01:14 \~ 01:16 Let's see what happens.
01:16 \~ 01:17 Is the patient relaxed?
01:17 \~ 01:18 The operator is coming soon.
01:18 \~ 01:23 100 Rocuronium given after induction.
01:23 \~ 01:26 The patient is presumably relaxed now.
01:26 \~ 01:28 The surgeon can come here.
01:28 \~ 01:28 Very good.
01:28 \~ 01:31 Let's assume surgery has started.
01:31 \~ 01:32 I'll adjust positioning a bit.
01:32 \~ 01:33 That's good.
01:33 \~ 01:34 What are you doing?
01:34 \~ 01:36 Positioning.
01:36 \~ 01:37 I am the positioning nurse.
01:37 \~ 01:38 Alright.
01:38 \~ 01:41 Nice to see you again.
01:41 \~ 01:43 I'm back after a break.
01:45 \~ 01:48 I'm doing another positioning now.
01:48 \~ 01:49 You can stay lying down.
02:08 \~ 02:15 He is in a hurry.
02:15 \~ 02:18 Okay, then I'll finish positioning.
02:18 \~ 02:22 I'm writing down the vitals.
02:22 \~ 02:23 I'm new on the job.
02:23 \~ 02:28 Could you please... Keep the infusion running.
02:28 \~ 02:31 Head down?
02:31 \~ 02:31 No, head up.
02:37 \~ 02:42 How much head up do we do?
02:42 \~ 02:44 You have to tell me how much you want head up?
02:44 \~ 02:45 Yeah, that's totally up to you.
02:45 \~ 02:48 Oh no, now the... Oh, oh, oh.
02:48 \~ 02:49 It slipped!
02:49 \~ 02:53 Sorry, I did not hold it during moving.
02:53 \~ 02:53 Okay.
02:53 \~ 03:09 I'm not sure how this fits with the scenario
03:09 \~ 03:13 Maybe the Voice of God can give us some input here.
03:16 \~ 03:20 Sorry.
03:26 \~ 03:27 I'm a clumsy man.
03:27 \~ 03:30 Okay, can you please call the senior physician?
03:30 \~ 03:31 I'm calling the senior doctor.
03:31 \~ 03:33 Please call the senior doctor.
03:33 \~ 03:36 The tube is also out, my god.
03:42 \~ 03:45 Come on.
03:45 \~ 03:46 Yes, the senior doctor is coming.
03:49 \~ 03:53 I am "*name*", an assistant from the OR.
03:53 \~ 03:55 We have an airway problem and a circulation problem.
03:55 \~ 03:58 Please come.
03:58 \~ 04:00 We are in F-OR, room 6.
04:00 \~ 04:04 Room 6 is right, right?
04:04 \~ 04:09 The positioning nurse pulled the cannula and the tube.
04:09 \~ 04:10 We have no cannula and no airway.
04:10 \~ 04:13 We have an airway problem.
04:13 \~ 04:14 Who is at the head?
04:14 \~ 04:17 I am at the head.
04:17 \~ 04:23 I switch propofol to gas and will ventilate a bit until we get a... I will do it well.
04:23 \~ 04:25 Can you ventilate?
04:25 \~ 04:26 I don't know.
04:26 \~ 04:28 Wait, I'm trying.
04:28 \~ 04:32 Please get the videolaryngoscope and tube on site.
04:32 \~ 04:33 Yes, sir.
04:33 \~ 04:35 Glidescope is ready.
04:35 \~ 04:39 Intubation was fiber-optically awake in bariatric surgery.
04:39 \~ 04:39 Okay.
04:39 \~ 04:40 D-Blade, does that suit you?
04:40 \~ 04:41 Can we ventilate?
04:41 \~ 04:42 I switch to 100% oxygen.
04:42 \~ 04:46 You cannot ventilate.
04:46 \~ 04:47 High resistance.
04:47 \~ 04:59 Okay, you do mask ventilation, I will do medication. Head position, get the bag.
04:59 \~ 05:03 Would you like to take over?
05:07 \~ 05:16 We will ventilate four-handed.
05:22 \~ 05:23 D-Blade is ready.
05:23 \~ 05:23 How is it?
05:23 \~ 05:27 Nothing goes in, no CO2.
05:27 \~ 05:28 D-Blade is there.
05:28 \~ 05:31 Listen, we cannot ventilate.
05:31 \~ 05:34 One intubation attempt, Guillermo.
05:34 \~ 05:36 Yes, sir.
05:36 \~ 05:40 And please get everything ready for the cricothyrotomy, Jonas.
05:40 \~ 05:41 Yes.
05:41 \~ 05:42 And we have placed 14G cannulas.
05:43 \~ 05:46 Are you monitoring circulation and anesthesia?
05:46 \~ 05:47 I'm giving the tube.
05:47 \~ 05:49 I'm intubating.
05:49 \~ 05:51 Will this work or not at all?
05:51 \~ 05:53 You can see nothing.
05:54 \~ 06:01 We cannot ventilate, we cannot intubate.
06:01 \~ 06:05 We need help from ENT now and we are doing a cricothyrotomy.
06:05 \~ 06:07 I'm calling ENT.
06:09 \~ 06:12 "*name*", please get the cricothyrotomy set.
06:12 \~ 06:15 Yes, I have it here.
06:17 \~ 06:20 I'll do the cricothyrotomy.
06:20 \~ 06:24 I have good exposure.
06:24 \~ 06:27 Please prepare the jet ventilation.
06:27 \~ 06:29 Jet ventilation is ready.
06:29 \~ 06:34 I'm doing the incision now.
06:34 \~ 06:36 Connect the ventilation.
06:37 \~ 06:40 We are ventilating again.
06:40 \~ 06:42 Excellent.
06:42 \~ 06:44 Good, good.
06:44 \~ 06:46 Patient is ventilated.
06:46 \~ 06:48 Patient is ventilated again.
06:48 \~ 06:51 We have an airway, excellent.
06:51 \~ 06:54 Circulation is stable again.
06:54 \~ 06:57 Very good.
06:57 \~ 07:00 We have oxygen.
07:00 \~ 07:03 This is our crisis management, well done everyone.
07:03 \~ 07:06 Great teamwork.
07:06 \~ 07:10 Thank you, everyone.
07:10 \~ End of scenario

Sapient AI vs. Chat GPT

Sapient AI | output | Chat GPT

Recognizing the Importance of Teamwork
SPEAKER_03 addresses task distribution: "Then you'll be leading anesthesia and we'll work along with that, right?" and SPEAKER_04 replies: "Just like in the usual case." This shows that team members understand their roles and work together.

Creating a Psychologically Safe Environment
SPEAKER_00 feels safe enough to admit being new to the job: "I'm new on the job."
SPEAKER_01 asks for help: "Can you please call the senior doctor?"
SPEAKER_00 admits a mistake: "I'm a clumsy man."

Structured Communication
SPEAKER_03 provides structured information about the patient: "He has a gastric bypass, which is next to be done. He's intubated."
SPEAKER_01 confirms the size of the venous access: "1.17-gauge."

Closed-Loop Communication
SPEAKER_01 confirms the medication: "100 given after induction."
SPEAKER_02 gives instructions for intubation: "Please set up the video laryngoscope and have the tube ready."
SPEAKER_01 confirms the action: "Yes."

Asking Clarifying Questions
SPEAKER_01 asks about the necessity of an action: "And why head down?"
SPEAKER_00 asks about the desired position: "How much head-up do we want?"
SPEAKER_02 asks about the status: "Can you ventilate?"

Sharing Unique Information
SPEAKER_03 shares specific information about the patient: "He has a 17-gauge cannula, located down there."
SPEAKER_01 gives details on medication: "They gave 0.2 fentanyl, 100 esmeron, and the propofol is running at 4."
SPEAKER_02 shares the plan for emergency intervention: "We now need help from ENT, and we're going to do a cricothyrotomy."

Optimizing Shared Mental Models
SPEAKER_03 gives an overview of the patient's condition: "We are intubated. For induction, he was fiberoptically awake."
SPEAKER_02 summarizes the situation: "We have an airway problem."
SPEAKER_02 gives a clear instruction: "Then palpate the Adam's apple, and cut down two centimeters."

Mutual Trust
SPEAKER_00 shows trust in the team by mentioning his return after a break: "It's nice to see you again. I'm back after some time off."
SPEAKER_04 trusts the instructions from SPEAKER_02: "ENT is informed, they'll send someone as soon as possible."

Mutual Performance Monitoring
SPEAKER_01 monitors medication: "I'm switching propofol to gas and will give a few extra breaths."
SPEAKER_02 monitors intubation: "Please set up the video laryngoscope and have the tube ready."
SPEAKER_02 checks the execution of the cricothyrotomy: "Stab incision, stab incision like this, horizontal."

Reflection and Debriefing
SPEAKER_02 reflects on the situation: "We have an airway problem."
SPEAKER_02 plans the next steps: "We will ventilate with four hands."
SPEAKER_02 summarizes the situation and plans further action: "We have a secured airway. Now let's do a quick 10 to 10."

1. Recognizing the Importance of Teamwork

The team demonstrates strong awareness of the importance of collaboration, especially in emergency situations:

"Who's at the head?" – "I'm at the head."
→ The clear role assignment shows that team members know and fulfill their responsibilities.

2. Creating a Psychologically Safe Environment

Examples show that team members can admit mistakes or ask questions:

"Oh no, now I've... Oh, oh, oh."
→ A team member openly admits a mishap.

"Is the patient relaxed?"
→ Shows that questions can be asked without fear of criticism.

3. Structured Communication

Clear and structured communication was often evident:

"We have an A problem and a C problem. Please come."
→ Clear naming of problems for precise feedback.

"Adam's apple palpation, cut down two centimeters."
→ Precise instructions during the cricothyrotomy.

4. Closed-Loop Communication

Messages were correctly received and confirmed:

"Please call the senior doctor." – "I'll call the senior doctor."
→ Clear order, executed and reported back.

"Can we ventilate?" – "No, we can't."
→ Precise feedback to critical questions.

"Do we have CO_2?" – "CO_2 is coming."
→ The reply confirms successful intervention.

5. Asking Clarifying Questions

Clarifying questions were used to avoid misunderstandings:

"How much head-up do we want?"
→ Clarification when instructions were unclear.

"With D-Blade, does that work for you?"
→ Clarifying equipment choice.

6. Sharing Unique Information

Critical information was shared in context:

"We have bradycardia."
→ Early warning about the circulatory situation.

"The intubation was awake fiberoptically during bariatric surgery."
→ Background information to guide appropriate action.

7. Optimizing Shared Mental Models

The team demonstrated a shared understanding:

"We're doing a cricothyrotomy now."
→ Clear agreement before a critical measure.

"Let's go through everything: A, B, C."
→ The checklist secures the shared understanding of the situation.

8. Mutual Trust

Trust in the team was evident:

"I'm intubating. Will it work?" – "You can do it."
→ Encouragement in a critical situation.

"Can you ventilate?" – "Wait, I'll try."
→ Open communication without fear of failed attempts.

9. Mutual Performance Monitoring

The team monitored each other and provided support:

"I'm switching from propofol to gas and will give a few extra breaths."
→ Collegial support with ventilation.

"Can you finish the rest, please?"
→ Tasks were delegated to increase efficiency.

10. Reflection and Debriefing

Reflection was evident:

"Let's go through everything: A. We performed a coniotomy."
→ Systematic review after the procedure.

"We now somehow need to go from the coniotomy back to the tube."
→ Forward planning of the next steps.

"Let's do a bronchoscopy to have a look from above."
→ Reflection and strategy adjustment based on prior observations.
